# *Kdr* genotyping (V1016I, F1534C) of the Na_v_ channel of *Aedes aegypti* (L.) mosquito populations in Harris County (Houston), Texas, USA, after Permanone 31–66 field tests and its influence on probability of survival

**DOI:** 10.1371/journal.pntd.0009833

**Published:** 2021-11-04

**Authors:** Jonathan R. Hernandez, Michael Longnecker, Chris L. Fredregill, Mustapha Debboun, Patricia V. Pietrantonio

**Affiliations:** 1 Department of Entomology, Texas A&M University, College Station, Texas, United States of America; 2 Department of Statistics, Texas A&M University, College Station, Texas, United States of America; 3 Harris County Public Health, Mosquito and Vector Control Division (HCPH-MVCD), Houston, Texas, United States of America; Faculty of Science, Mahidol University, THAILAND

## Abstract

*Aedes aegypti* (L.) is an important mosquito vector of emerging arboviruses such as Zika, dengue, yellow fever, and chikungunya. To quell potential disease outbreaks, its populations are controlled by applying pyrethroid insecticides, which selection pressure may lead to the development of insecticide resistance. Target site insensitivity to pyrethroids caused by non-synonymous knockdown resistance (*kdr*) mutations in the voltage-gated sodium (Na_V_) channel is a predominant mechanism of resistance in mosquitoes. To evaluate the potential impact of pyrethroid resistance on vector control, *Ae*. *aegypti* eggs were collected from eight mosquito control operational areas in Harris County, Texas, and emerged females were treated in field tests at four different distances from the pyrethroid Permanone 31–66 source. The females were genotyped by melting curve analyses to detect two *kdr* mutations (V1016I and F1534C) in the Na_V_ channel. Harris County females had higher survivorship rates at each distance than the pyrethroid-susceptible Orlando strain females. Survivorship increased with distance from the pyrethroid source, with 39% of field-collected mosquitoes surviving at 7.62 m and 82.3% at 22.86 m from the treatment source. Both the V1016I and F1534C pyrethroid resistant genotypes were widely distributed and at high frequency, with 77% of the females being double homozygous resistant (II/CC), this being the first report of *kdr* mutations in *Ae*. *aegypti* in Harris County. Analysis of the probability of survival for each mutation site independently indicated that the CC genotype had similar probability of survival as the FC heterozygous, while the II genotype had higher survival than both the VI and VV, that did not differ. The double homozygous resistant genotype (II/CC) had the highest probability of survival. A linear model estimated probability of survival for areas and genotypes. The high frequency and widespread distribution of double-homozygote pyrethroid-resistant *Ae*. *aegypti* may jeopardize disease vector control efforts in Harris County.

## Author summary

Females of the mosquito *Aedes aegypti* transmit arboviruses of human diseases including Zika, dengue, chikungunya, and yellow fever, and *Culex quinquefasciatus* transmits West Nile virus (WNV). Upon WNV detection in Harris County, TX, Harris County Public Health Mosquito and Vector Control Division applies pyrethroid or organophosphate insecticides to control *Cx*. *quinquefasciatus* mosquitoes. Pyrethroids target the sodium channel and knockdown resistance (*kdr)* mutations in this channel confer resistance to pyrethroids and increase mosquito survival. While *Ae*. *aegypti* is not a current target for public mosquito control in Harris County, we investigated frequency and spatial distribution of two *kdr* mutations in *Ae*. *aegypti* females, and their impact on pyrethroid survival after field tests were conducted with Permanone 31–66. The survival probability of mosquitoes varied depending on distance from application, and on their genotype and area of origin. Survivorship was lowest at 7.62 m, but most females survived at and beyond 15.24 m. Most (98.3%) females genotyped carried at least one *kdr* allele, and 77% were double homozygous resistant (II/CC). This is the first report of these resistant genotypes in Harris County. The resistant genotype II/CC had the highest survival probability. These results should be useful when modeling the risk of mosquito-borne diseases.

## Introduction

*Aedes aegypti* (L.) is the most successful primary vector of emerging and reemerging arboviral diseases which include Zika, dengue, chikungunya, and yellow fever [1]. This species is also a competent vector for the emerging Mayaro virus (MAYV), which has caused outbreaks in Europe and the Americas [2,3]. Concern for MAYV spread has grown due to demonstrated infections in sick travelers and migratory birds [4,5].

The worldwide distribution of *Ae*. *aegypti* mosquitoes and their adaptation to anthropogenic environments has made them one of the greatest threats to global human health [6]. Changes in urbanization patterns and climate can influence the distribution of *Ae*. *aegypti*, which in turn increases the potential for mosquito-borne disease transmission [7,8]. A tetravalent live attenuated dengue vaccine CYD-TDV (Dengvaxia) features a chimeric dengue-yellow fever virus 17D (YF17D), however, the WHO recommends vaccination only in DENV-seropositive populations due to risk of adverse effects [9]. Therefore, the primary method of controlling mosquito-borne diseases spread by *Ae*. *aegypti* still focuses on controlling adult mosquitoes, often done through insecticide application, and it can be made more sustainable through integrated mosquito management during epidemic seasons [10].

Pyrethroids are one of the most widely applied insecticides by public vector control programs, private pest control operators and homeowners as they are highly effective in the field and have low toxicity to mammals. Pyrethroids, such as permethrin and deltamethrin, modulate the Na_V_ channel by binding to its open conformation and preventing channel closure [11]. This binding prolongs action potentials, resulting in paralysis and fast insect “knockdown” [12]. However, the frequent and widespread use of pyrethroids has resulted in resistant mosquito populations worldwide, diminishing the long-term effectiveness of vector and disease control strategies [13]. The ever-growing human population and increasing urbanization necessitates more frequent pyrethroid treatments in high-density urban areas, which likely contributes to the global increase in pyrethroid resistance in mosquito populations [14,15].

Target site insensitivity to pyrethroid insecticides caused by single-point non-synonymous mutations in the Na_V_ channel is called “knockdown resistance” or *kdr* [16]. These *kdr* mutations are phenylalanine at position 1534 to cysteine (F1534C; codon change from Phe (T**T**C) to Cys (T**G**C) [17]), and valine at position 1016 to isoleucine (V1016I; codon change from Val (**G**TA) to Ile (**A**TA) [18]). The F1534C and V1016I *kdr* mutations are associated with resistance to pyrethroid-based insecticides in *Ae*. *aegypti* [19], and both mutations have been detected in *Ae*. *aegypti* in the United States (US) [20,21]. *Ae*. *aegypti* mosquitoes homozygous for the F1534C mutation (1534C:ROCK strain) have resistance to type I (i.e. permethrin) and type II pyrethroids (i.e. deltamethrin), and *in vitro*, double mutant channels of the AaNa_v_1-1 (V^1016^I+F^1534^C) are resistant to deltamethrin and more resistant to permethrin compared to channels with the F^1534^C alone [22,23]. The co-occurrence of these *kdr* mutations is common in *Ae*. *aegypti* outside of Texas, with as high as 90% frequency in some municipalities in Central and South America, likely due to strong selection caused by repeated pyrethroid use [24,25].

Harris County is the third most populous county in the US with approximately 4.71 million residents, and includes Houston, the fourth largest city in the US [26]. To protect the health of Harris County’s residents from arboviruses, Harris County Public Health, Mosquito and Vector Control Division (HCPH-MVCD) monitors mosquito traps in 268 operational areas within the mosquito surveillance program. They apply ultra-low volume (ULV) adulticide treatments against *Culex quinquefasciatus* (Say) mosquitoes, the most abundant local species, when arboviruses such as St. Louis Encephalitis or West Nile virus (WNV) are detected in Harris County populations.

To date, the HCPH-MVCD has not deployed targeted adulticide-based control for *Ae*. *aegypti* or *Aedes albopictus*, despite both species being present in this region, but they would be targeted if dengue, chikungunya, or Zika viruses were detected. Chemical control for *Ae*. *aegypti* mosquitoes in residential areas would be conducted by applying Permanone 31–66 (active ingredient (a.i.) 31% permethrin; synergist, 66% piperonyl butoxide (PBO)) because this is the only approved insecticide that can be applied by an operator with a ULV hand-held sprayer to reach distances up to 15.24 meters [27]. Treatment for *Cx*. *quinquefasciatus* is done with alternate applications of Permanone 31–66 and malathion (an organophosphate) through a truck fleet equipped with London Fog Model 18–20 high output ULV aerosol generators that are designed to reach further distances, approximately 30.48 meters. Since Harris County frequently applies high doses of ULV pyrethroids to treat *Cx*. *quinquefasciatus*, there is concern that local *Ae*. *aegypti* populations may have developed pyrethroid resistance through selection for *kdr* mutations [28].

In Harris County there is little information on the frequency of the Na_v_ channel *kdr* mutations that confer pyrethroid resistance in *Ae*. *aegypti*. The only such study found that all mosquitoes tested were homozygous for the wild-type V1016 and F1534 genotypes and therefore susceptible to pyrethroids [25]. However, the mosquitoes used in that study were collected in 1999, and there is now a 20-year gap in our knowledge of the *kdr* resistance mutation status in Harris County *Ae*. *aegypti* populations.

The objectives of this study were to determine: first, the susceptibility of *Ae*. *aegypti* females collected in eight operational areas of Harris County to Permanone 31–66 in field cage tests, where in each test cages were placed at three distances from the applicator path, and second, estimate the frequency of the *kdr* mutations, V1016I and F1534C, in surviving and dead mosquitoes from these tests. Mutations in the Na_v_ channel were analyzed by diagnostic melting curve analyses to obtain updated *kdr* frequencies to help inform future *Ae*. *aegypti* control efforts.

This is the first report of Na_v_ channel target site insensitivity mutations conferring pyrethroid resistance in *Ae*. *aegypti* mosquito populations in Harris County, while simultaneously evaluating the effectiveness of pyrethroid-based vector control under field conditions and determining genotype survival probabilities. This information is crucial in view of the more often suitable climatic conditions favoring increasing mosquito population densities and associated disease transmission, as this area has recently experienced several hurricanes that were previously classified as 500-year events [29,30].

## Materials and methods

### Mosquito collection and rearing

**Fig 1:**
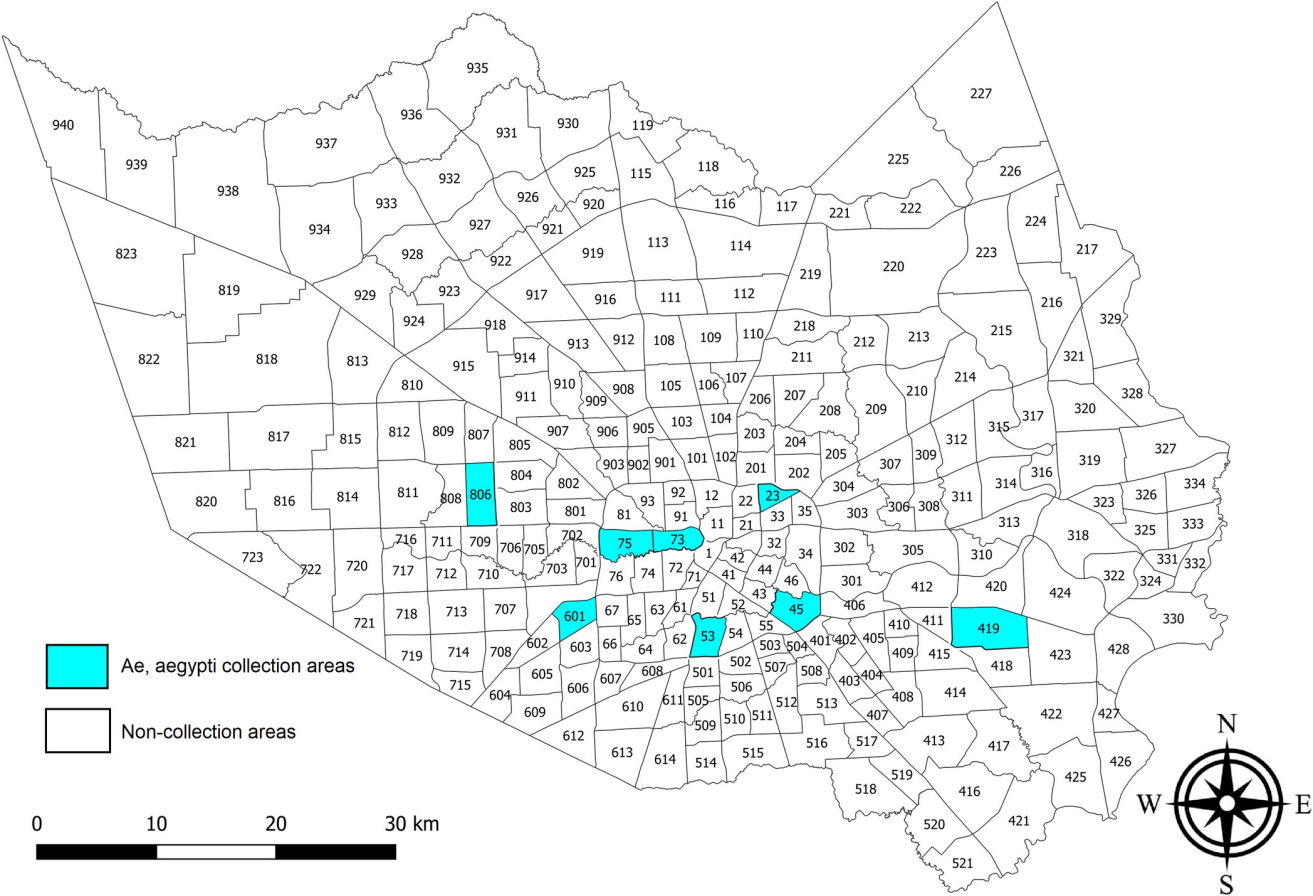
Map of 268 mosquito and vector control operational areas in Harris County. Eight areas (marked in turquoise) were selected for *Aedes aegypti* sampling out of the 268 HCPH-MVCD operational areas. From west to east, these were: 806, 601, 75, 73, 53, 23, 45, and 419. The areas of origin of mosquitoes and test dates were: Area 23, November 9, 2017; Area 419, September 9, 2018; Area 53, October 11, 2018; Area 73, November 6, 2018; Areas 45 and 75, July 16, 2019; Areas 601 and 806, August 6, 2019. This map was generated with the Harris County boundary (map image layer by PHES_AGO; created Jun 7, 2017, updated May 7, 2020) and operational area boundaries (map image layer by PHES_AGO; created Nov 4, 2016, updated May 9, 2020) using QGIS 3.12 software (www.qgis.org). The layers for county (Map service: Harris County boundary masked) (https://www.arcgis.com/home/item.html?id=a8aa2ef4067348c79ccea62857a2f623) and for Harris County operational area boundaries (MVCDOperational_Areas) (https://www.arcgis.com/home/item.html?id=66643535e01b42d3aae5d4647f5e1a6c) were created by Harris County Public Health and are publicly available. There are no special restrictions or limitations on the terms of use of the layers applied to this map.

Eight mosquito and vector control operational areas within Harris County, Texas, served by HCPH-MVCD were chosen for mosquito field-cage bioassays to evaluate the efficacy of pyrethroid adulticides against *Aedes aegypti* females (Fig 1). During the fall of 2017, 2018, and summer and fall of 2019, wild *Ae*. *aegypti* eggs from these areas were collected in black plastic cups (473 ml; RushKing Promotions, Brooklyn, NY) that were filled with tap water and included a trifold paper towel to act as oviposition substrate (S1A Fig). The HCPH-MVCD deployment teams set these cups in their assigned areas during early morning and collected the cups and eggs every third morning. The eggs were placed in plastic bags for transport to the laboratory and the estimated number of eggs collected from each area site was recorded (S1B Fig). The eggs were brought to the HCPH-MVCD insectary, where they were hatched and reared in the laboratory at 28–29.4°C. After adult emergence, two- to five-day-old females were separated from males using an aspirator to obtain the first adult generation to be field-tested. The field bioassays required an average of 25 females for each of the 12 cages per area, as discussed later in the field cage tests section.

In the laboratory, the collected eggs were placed into water-filled pans for hatching. Their incubation was synchronized with that of eggs from the Orlando susceptible laboratory strain (S1C Fig) to produce adults of the same age for field tests. After the eggs hatched, the first instar larvae were fed 1.25 ml of a flurry of ground tropical flake fish food (Tetra Holding, Inc., Blacksburg, VA) and feeding was repeated at the start of the third larval instar. Pupae were placed in emergence dishes, and these were placed into adult cages together with adult feeders containing a solution of sucrose (10%) (S1D Fig). Following adult eclosion, the emergence dishes were removed two days prior to the field cage testing to ensure all female mosquitoes used in the tests were minimally 2 days-old and up to 5 days old post-emergence. The wild *Ae*. *aegypti* females obtained were separated manually from any contaminant *Ae*. *albopictus* females in the rearing cages by identifying their distinct thorax markings: a lyre-like figure in *Ae*. *aegypti* and straight central line in *Ae*. *albopictus* [31]. Females used in field tests were virgin and non-blood fed. Females were selected for the field tests because only females blood feed on humans and are the vectors for arboviruses, and while they also feed on nectar [32], males feed exclusively on sugar-containing solutions such as nectar from plants.

### Field cage tests

**Fig 2:**
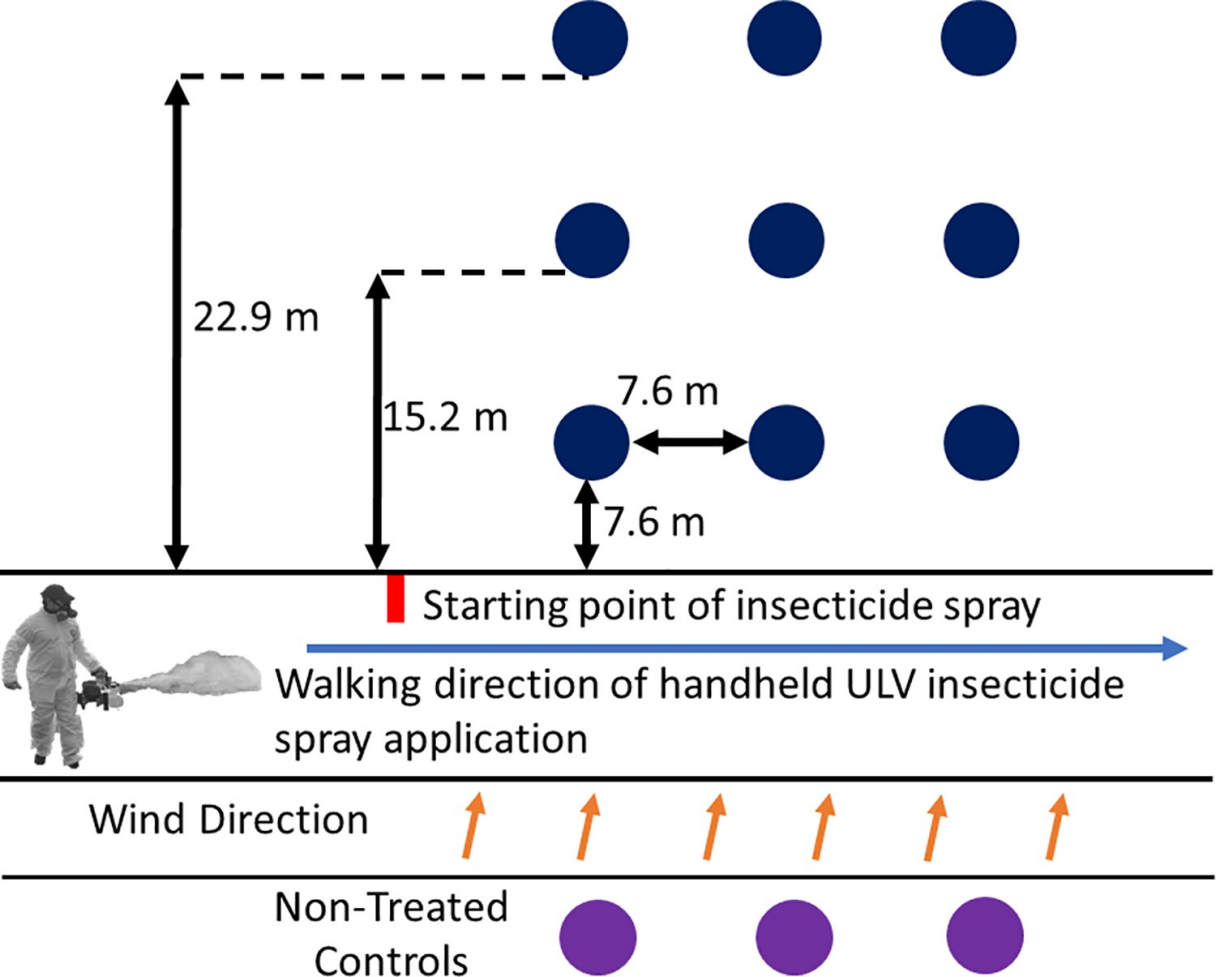
Schematic of *Aedes aegypti* field cage test for effectiveness of Permanone 31–66 treatment. Blue and purple dots represent a post with cages containing females for testing mosquitoes from operational areas, that were positioned at distances 7.62, 15.24, and 22.86 m from the application path in which the person applying the insecticide walks (~2 mph) with a Colt-4 ULV Handheld Fogger Sprayer directed towards the posts. Three posts were placed at each distance from the path. Area 23 used posts placed at 7.62 m, 22.86 m, and 38.1 m from the application path. Posts represented by purple dots serve as negative controls, being unexposed to Permanone 31–66.

The field cage test methodology for detecting susceptibility of *Ae*. *aegypti* to permethrin was modified from Stark et al. [33] (Fig 2). Tests within the same year were conducted at approximately three-week intervals using mosquitoes from different operational areas, in accordance with Harris County’s field cage testing program schedule. Field tests were conducted between November 2017 and August 2019, and the areas of origin of mosquitoes and test dates were: Area 23, November 9, 2017; Area 419, September 9, 2018; Area 53, October 11, 2018; Area 73, November 6, 2018; Areas 45 and 75, July 16, 2019; Areas 601 and 806, August 6, 2019. These areas were selected based on previous field surveys and historical mosquito surveillance utilizing BG-Sentinel traps that confirmed *Ae*. *aegypti* presence in these areas, as described by Dennett et al. [34]. In total, 1,016 *Ae*. *aegypti* females from Harris County operation areas, and 1,738 pyrethroid-susceptible Orlando strain females as controls were treated with Permanone 31–66.

The field cage test was designed to evaluate Permanone 31–66 effectiveness at three distances from the ULV insecticide manual application treatment, simulating real mosquito control scenarios [28]. The typical field test plot was a 3 x 3 design with a row of three cage posts positioned at 7.62, 15.24, and 22.86 meters downwind from the insecticide application (Fig 2), and within each distance, three cage-carrying posts were positioned at 7.62 meters from each other as pseudoreplicates. The first assay was that of area 23 in 2017 and used distances at 7.62, 22.86, and 38.1 m, as 38.1 m is the maximal expected application swath when using the Colt-4 Fogger outdoors [27]. This latter distance was not tested in subsequent assays. The minimal distance of 7.62 meters was chosen based on the average size of a house yard in Houston [35]. The nine posts with cages were placed downwind to receive the insecticide treatment and three similar posts with cages were placed upwind from the treatment source as negative controls. These mosquitoes did not receive insecticide treatment, thus, any mortality that occurred was considered as a baseline and used to correct the mortality in the treated cages using the Abbott’s formula [36]. Females of the Orlando strain served as positive controls through their inclusion in one cage per pole in the treatment plot, and as negative controls through their placement upwind of the insecticide application. Females obtained from one rearing cycle from the Harris County operational areas chosen to be tested were placed in at least two cages per post and in three cages in most posts (Fig 2). In 2019 field cage tests, three cages were used per post, one with the Orlando strain and two with females from two operational areas. All tests used a permethrin application rate of 0.008 kg (ai)/ha, the recommended maximum application rate. A test is defined as a single-pass of an operator (man)-held Colt-4 ULV Handheld Fogger Sprayer (London Fog, Minneapolis, MN) applying Permanone 31–66, with the direction of the pesticide treatment being perpendicular to the walking path (Fig 2); this is a cold fogger that produces droplets of less than 15 μm, which is considered optimal for *Ae*. *aegypti* control [37]. A typical test used approximately 350 female mosquitoes, of which 20% were of the Orlando strain.

The test location was an open field located at HCPH-MCVCD. Assays were conducted during the early morning and up to 10 am when a reasonably consistent wind direction toward the cages to be treated was achieved. Weather information (temperature, relative humidity, and wind speed) was continuously obtained, before and throughout each test, using a hand-held Kestrel Pocket Weather Meter (Model 4000, Nielsen Kellerman, Boothwyn, PA), and with wind direction was roughly estimated using a compass and a ribbon to catch wind.

### Genomic DNA extraction

Genomic DNA was extracted from 826 *Ae*. *aegypti* females from the natural populations collected in Harris County and from the reference insecticide-susceptible Orlando strain females. DNA was isolated from whole bodies of individual mosquitoes using the ZYMO Quick-DNA Microprep kit, following the manufacturer’s instructions except that a one-hour incubation period in the DNA lysis buffer was used to obtain greater DNA yields. The concentration and quality of each DNA sample were determined using an automatic microplate reader (Tecan Infinite M200). Genomic DNA from individual mosquitoes was then diluted to 15 μL aliquots (10 ng/μL) and stored at -80°C until diagnostic PCR was performed to detect the *kdr* mutations.

### Genomic DNA analyses

DNA melting curve analyses of allele-specific PCR products were carried out in 96-well plates using a QuantStudio 6 Pro system (Applied Biosystems, Foster City, CA) and diagnostic primers for the V1016I and F1534C mutations in the Na_v_ channel (Table 1). All reactions were performed in triplicate as technical replicates. Peaks were analyzed using Thermo Fisher’s Design and Analysis v 2.3 software (Thermo Fisher Scientific Inc, Waltham, MA, US). This test determined the genotypes of the females at the V1016I site as previously described by Saavedra-Rodriguez et al. [18], with the exception of using an improved common reverse primer from Pinto et al. [38]. Melting curve analyses for the F1534C were as reported by Yanola et al. [17].

**Table 1:** Sequences of primers used for *kdr* mutations detection in the *Aedes aegypti* Na_v_ channel.

*Kdr* Mutation	Primer Name	Primer Sequence (5’ -> 3’)
V1016I^a^	Val1016f	[GCGGGCAGGGCGGCGGGGGCGGGGCC]ACAAATTGTTTCCCACCCGCAC*C*G**G**
	Iso1016f	[GCGGGC]ACAAATTGTTTCCCACCCGCAC*T*G**A**
	Iso1016-r^c^	TGATGAACCSGAATTGGACAAAAGC
F1534C^b^	F1534f	[GCGGGC]TCTACTTTGTGTTCTTCATCAT*A*T**T**
	C1534f	[GCGGGCAGGGCGGCGGGGGCGGGGCC]TCTACTTTGTGTTCTTCATCAT*G*T**G**
	F1534r	TCTGCTCGTTGAAGTTGTCGAT

Primers feature base pair mismatches introduced at the third base from 3’ end to increase allele specificity (italics); the diagnostic differential nucleotide is in bold, underlined. For Val 1016 to Ile (Iso) the diagnostic nucleotide is the first in the codon (**G**TA to **A**TA) while for F1534C is the second nucleotide of the codon (T**T**C or T**T**T to T**G**A or T**G**C).

a. Saavedra-Rodriguez et al. [18]

b. Yanola et al. [17]

c. Pinto et al. [38]

For the V1016I mutation detection, the 10 μL reaction mixture contained 5 μL of PowerUP SYBR Green Master Mix (Thermo Fisher Scientific, MA), 0.2 μM of the Val1016f and Iso1016r primers from Pinto et al. [38], 0.17 μM of the Iso1016f primer used by Saavedra-Rodriguez et al. [18], and 10 ng of genomic DNA (gDNA) template. PCR conditions consisted of a 3 min 95°C activation step, followed by 35 cycles at 95°C for 10 s, 60°C for 10 s, and 72°C for 30 s, with a final extension step of 15 s at 95°C. The melting curves were determined from 65°C to 95°C, with an increase of 0.2°C every 1 s. Based on the melting curves, a single peak at 78°C corresponded to the 82 bp homozygous resistant mutant (I1016/I1016), a peak at 86°C corresponded to the 102 bp homozygous susceptible wild-type (V1016/V1016), and two peaks, one at each of both temperatures corresponded to a heterozygous mutant (V1016/I1016).

For detection of the F1534C mutation, the 10 μL reaction mixture contained 5 μL of PowerUP SYBR Green Master Mix, 0.3 μM of each of the F1534f and F1534r primers, 0.33 μM of the C1534f primer, and 10 ng of the gDNA template. PCR conditions used included an activation step of 3 min at 95°C, followed by 35 cycles at 95°C for 10 s, 57°C for 30 s and 72°C for 30 s, with a final extension step of 10 s at 95°C. The melting curves were determined from 65°C to 95°C, with an increase of 0.1°C every second, where a single peak at 85°C corresponded to the 113 bp homozygous resistant mutant (C1534/C1534), a single peak at 80°C corresponded to the 93 bp homozygous susceptible wild type (F1534/F1534), and two peaks, one at each temperature, corresponded to the heterozygous resistant (F1534/C1534).

Each PCR reaction for a Harris County mosquito female was run in parallel with an Orlando strain female as a control, with primers to detect both wild-type susceptible genotypes (V1016/V1016 and F1534/F1534), which were run in separate reactions.

### Statistical analyses

Statistical analyses were performed using SAS 9.4 (Cary, NC) and GraphPad Prism (San Diego, CA). A Pearson Chi-Square test was used to compare the survivorship of mosquito females from different operational areas and treatment distances in the field cage test. If the *P*-value was lower than 0.05, the data were analyzed using a Bonferroni 2-sample proportions test or the Bonferroni corrected Fisher’s Exact test to determine significant differences between pairs of operational areas and distances used in the field cage tests [39]. The Chi-Square test and a Bonferroni corrected 2-sample proportions test or Bonferroni corrected Fisher’s Exact test were also applied to the analyses of the proportion of the individual (for F1534C: FF, FC, CC; for V1016I: VV, VI, II) and co-occurring *kdr* genotypes (VV/FF, VV/FC, VV/CC, VI/FF, VI/FC, VI/CC, II/FF, II/FC, II/CC) from the eight areas, overall allele frequencies (V, I, F, C) and the proportion of the genotypes in the surviving mosquitoes. The probability that genotypes affected female survivorship was assessed using the Cochran-Mantel-Haenszel (CMH) test statistic that adjusts for differences between the areas and distances used [40].

The impact of the factors 1) area, 2) genotype, 3) distance, and 4) cage within each distance on the probability of survival of the mosquitoes was assessed through a logistic regression model. The model of the log-odds, where p equals probability of survival, is:

$$log\left(\frac{p}{1-p}\right)=bo+b1*area+b2*genotype+b3*distance+b4*cage+e$$

An asymptomatic Wald’s procedure was used to obtain the confidence intervals (CI) for the probability of survival.

## Results

### Field cage test bioassays

The field cage tests evaluated if female survivorship differed among the eight operational areas (Fig 1) at each of three distances after Permanone 31–66 treatment with a Colt-4 ULV Handheld Fogger. The mortality data from areas 23 and 419 required correction with the Abbott’s formula [36] because two of the negative control females (Orlando strain) died (Fig 2). Mortality from the remaining tests did not require this correction.

**Fig 3:**
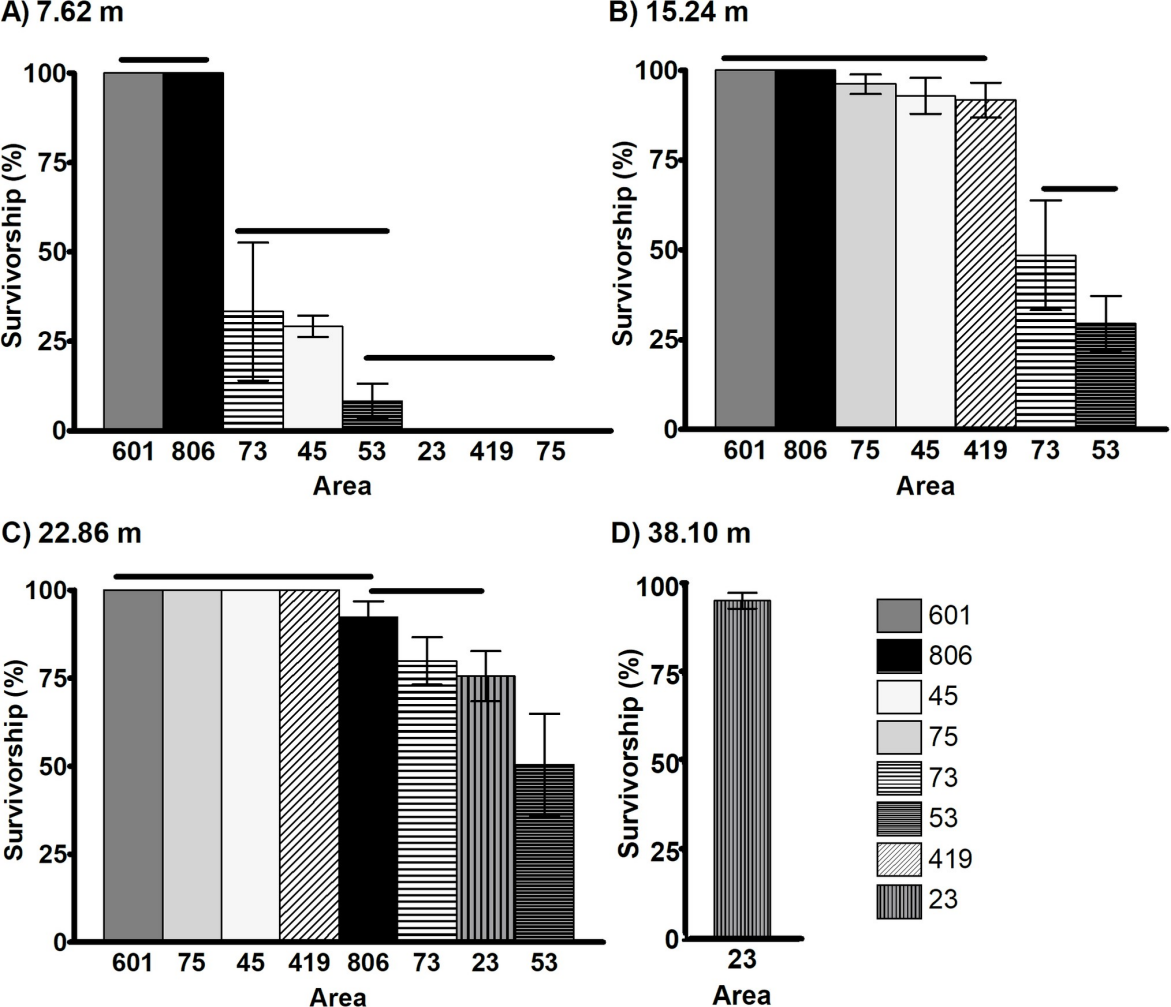
Survivorship percentage of *Ae*. *aegypti* in field tests from eight operational areas at different distances. For each distance, areas are organized in order of decreasing survivorship. Permanone 31–66 was applied at (A) 7.62 m from cages; (B) 15.24 m; (C) at 22.86 m; or (D) 38.1 m. There were significant differences in the survival rates among areas at each distance (Chi-Square Test; *P* < 0.0001 for each of the panels A-C). (D) Only one area was tested, thus, no Chi-square test could be performed for 38.1 m. The analyses were followed by pairwise comparison tests with Bonferroni correction to detect significant differences among areas within each distance. Horizontal lines above bars indicate the areas which are not significantly different (*P* < 0.05). For each area, histograms show Mean ± SD. The survivorship percentages shown were corrected for the mortality of the upwind Orlando strain mosquitoes for areas 23 and 53 in the respective assays.

When comparing survivorship among different areas and distances, mosquitoes from areas 601 and 806 had the highest survivorship of all areas tested (Fig 3A–3C). At 7.62 m, the survivorship of females from the eight areas was significantly different (Chi-square; *P* < 0.0001; Fig 3A). Pairwise comparisons between the areas indicated that females from areas 601 and 806 had similar survivorship which was significantly higher (>95%) than that from all other areas. Areas 73, 45 and 53 followed in survivorship, and were similar among themselves, while area 53 was intermediate in survivorship. Areas 73 and 45 differed from areas 23, 419, and 75, all of which that had the lowest survivorship (Fig 3A). At 15.24 m from the Permanone 31–66 application, survivorship among the eight areas was also significantly different (Chi-square; *P* < 0.0001). Survivorship in areas 601, 806, 75, 45, and 419 was similar and significantly higher when compared to survivorship in areas 73 and 53, with these latter two not significantly different from each other (Fig 3B). At 22.86 m, survivorship was also statistically different when comparing all areas (Chi-square; *P* < 0.0001) (Fig 3C). Females from areas 601, 75, 45, 419, and 806 had similar and significantly higher survivorship (>95%) than areas 73 and 23, while these latter two areas were not significantly different in survivorship from area 806. Area 53 had the lowest survivorship at about 50% and was different from all others (Fig 3C). At 38.1 m, females from area 23 had 96% survivorship (Fig 3D).

To evaluate the field control efficacy of Permanone 31–66, it is also critical to analyze the mortality of the downwind control susceptible mosquitoes treated simultaneously with those from the investigated areas. The susceptible Orlando (Or) treated controls were killed (> 98.8%) at 7.62 m in five of the six tests performed on different dates, indicating excellent Permanone 31–66 control (S2A Fig). The exception was that 14% of Orlando females survived in the August 6, 2019, test in which females from areas 601 and 806 had 100% survival, and these differences in survivorship were highly significant (S2A Fig). There was a higher and increasing survivorship of both control Orlando females and field-collected females with increasing distance in the treatment zones at 15.24 and 22.86 m, and both these distances had high variability in survivorship (S2B and S2C Fig). Despite this variability, all Orlando strain females were killed in three of the six tests at 22.86 m (November 9, 2017; September 6, 2018; October 11, 2018).

For the mosquitoes from operational areas, survivorship was almost 100% for areas 601 and 806 at the first three distances tested (S2A–S2C Fig), and at 15.24 and 22.86 m survivorship was also above 75% for areas 45 and 75, however, the Orlando mosquitoes treated simultaneously at 15.24 m died at a significantly higher rate (S2B Fig), indicating that the pesticide had reached that distance. Finally, for 22.86 m, mosquitoes from area 419 had 100% survivorship, while the susceptible strain controls died at significantly higher rates compared to females from areas 23, 419, 53, 45, and 75 (S2C Fig).

**Fig 4:**
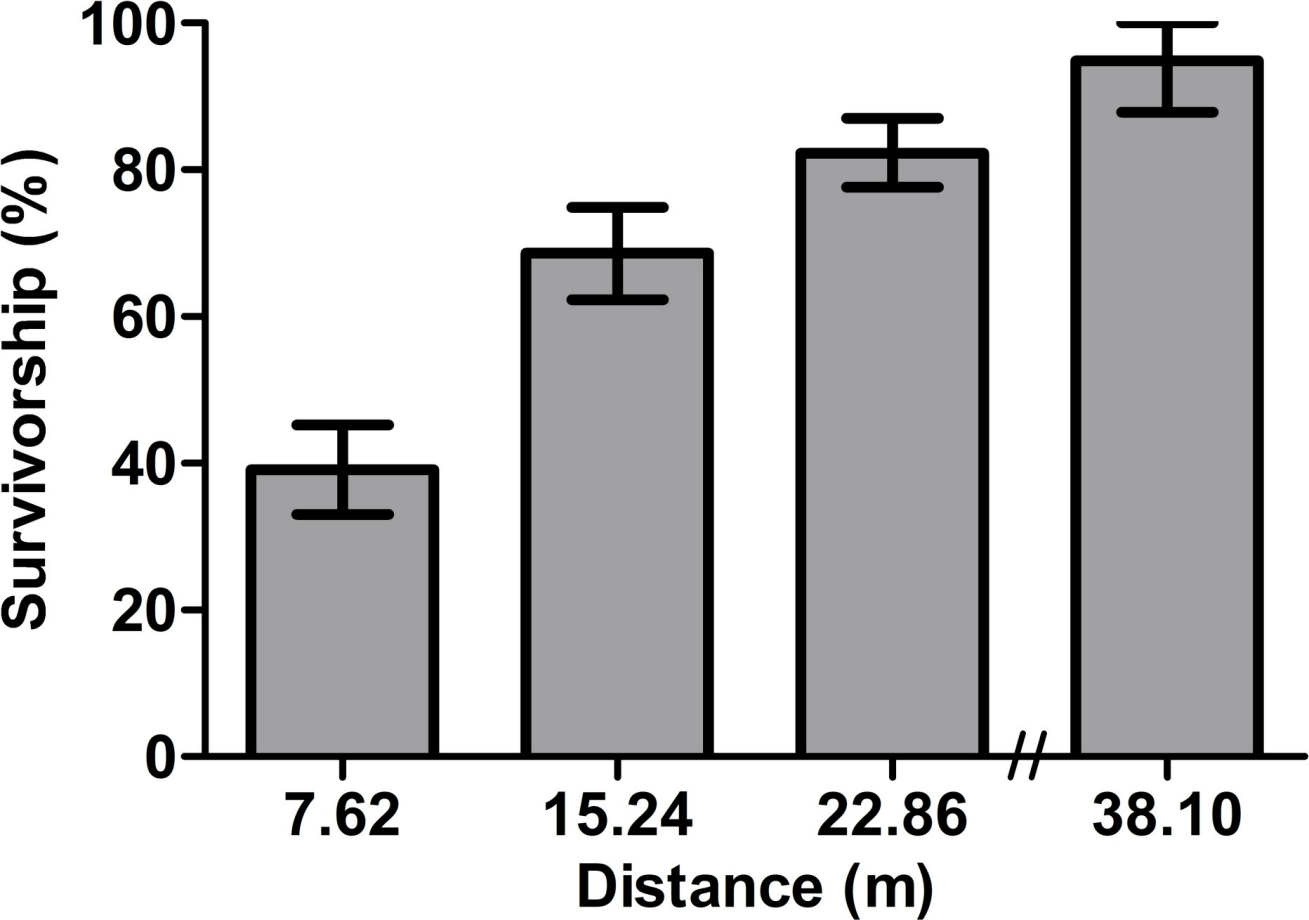
Overall survivorship percentage at each distance after Permanone 31–66 application. There were significant differences in female survivorship among the different distances (Chi-square Test; *P* < 0.0001). Females had significantly different probability of survivorship for all pairs of distances contrasted, based on a Bonferroni corrected 2-sample proportions test (*P* < 0.05); histogram shows Percentage ± CI.

To determine the overall effectiveness of Permanone 31–66 considering distance from the treatment application path, the survivorship of field collected females at each distance was combined for all areas. There was a statistically significant increase in survivorship for the field-collected mosquitos as the distance from the Permanone 31–66 treatment increased (Chi square, *P* < 0.0001) (Fig 4). The subsequent comparison of percent survivorship (Bonferroni corrected 2-sample proportions test) detected significant differences between the percent survivorship for all pairs of distances. Therefore, irrespective of operational area, control efficacy was the highest at 7.62 m, however incomplete, with a mean survivorship of 39% +/- 6% (Fig 4). For field-collected mosquitoes, even the recommended distance of 15.24 m did not provide acceptable control as survivorship was 68.6% (Fig 4), and survivorship was 82.3% at 22.86 m and 94.9% at 38.1 m.

### Genotype analysis

At the distance of 15.24 m and beyond, the application provided poor control, however, due to the extreme differences in survivorship observed among the eight areas, specifically for tests at 7.62 m (Fig 3), the females were genotyped for *kdr* mutations to determine if high frequency of these pyrethroid resistant mutations in the population could explain differences in survivorship. In total, 746 *Ae*. *aegypti* females from all areas and Permanone 31–66 field tests were genotyped for the V1016I and F1534C *kdr* mutations in the Na_V_ channel, and the results are shown in Table 2. The number of females analyzed per area was considered adequate for population studies [21], ranging from 47 to 168 in this study, except for area 419 from which only 21 females were available (Table 2). High frequencies of the dual-*kdr* genotype (II/CC) were observed in all areas; this genotype representing 77.3% of all mosquitoes tested (Table 2 and S3 Fig). This genotype was followed in frequency by VI/FC (8.2%), VI/CC (5.5%), and VV/CC (4.7%) (Table 2). The remaining co-occurring genotypes (II/FC, II/FF, VV/FC, and VV/FF) were between 0.4% and 1.7% of all mosquitoes tested. None of the mosquitoes tested were of the VI/FF genotype (Table 2).

**Table 2:** Na_V_ channel genotypes detected at sites 1016 and 1534 from *Aedes aegypti* in Harris County.

Date	Area	Co-occurring Genotypes								
		II/CC	II/FC	II/FF	VI/CC	VI/FC	VV/CC	VV/FC	VV/FF	Total
		N; (%)	N; (%)	N; (%)	N; (%)	N; (%)	N; (%)	N; (%)	N; (%)	
**11/9/17**	**23**	63; (76.8)	0; (0.0)	4; (4.9)	2; (2.4)	8; (9.8)	3; (3.7)	1; (1.2)	1; (1.2)	82
**9/6/18**	**419**	20; (95.2)	0; (0.0)	0; (0.0)	0; (0.0)	0; (0.0)	0; (0.0)	0; (0.0)	1; (4.8)	21
**10/11/18**	**53**	89; (67.4)	0; (0.0)	0; (0.0)	9; (6.8)	11; (8.3)	9; (6.8)	5; (3.8)	9; (6.8)	132
**11/6/18**	**73**	117; (69.6)	0; (0.0)	0; (0.0)	12; (7.1)	19; (11.3)	12; (7.1)	6; (3.6)	2; (1.2)	168
**7/16/19**	**45**	28; (59.6)	0; (0.0)	0; (0.0)	1; (2.1)	16; (34.0)	2; (4.3)	0; (0.0)	0; (0.0)	47
	**75**	87; (100)	0; (0.0)	0; (0.0)	0; (0.0)	0; (0.0)	0; (0.0)	0; (0.0)	0; (0.0)	87
**8/6/19**	**601**	85; (79.4)	0; (0.0)	0; (0.0)	10; (9.4)	4; (3.7)	8; (7.5)	0; (0.0)	0; (0.0)	107
	**806**	88; (86.3)	3; (2.9)	0; (0.0)	7; (6.9)	3; (2.9)	1; (1.0)	0; (0.0)	0; (0.0)	102
**Total**		577; (77.3)	3; (0.4)	4; (.5)	41; (5.5)	61; (8.2)	35; (4.7)	12; (1.6)	13; (1.7)	**746**

N = number of females (%) = percentage of females of the total for each area

**Fig 5:**
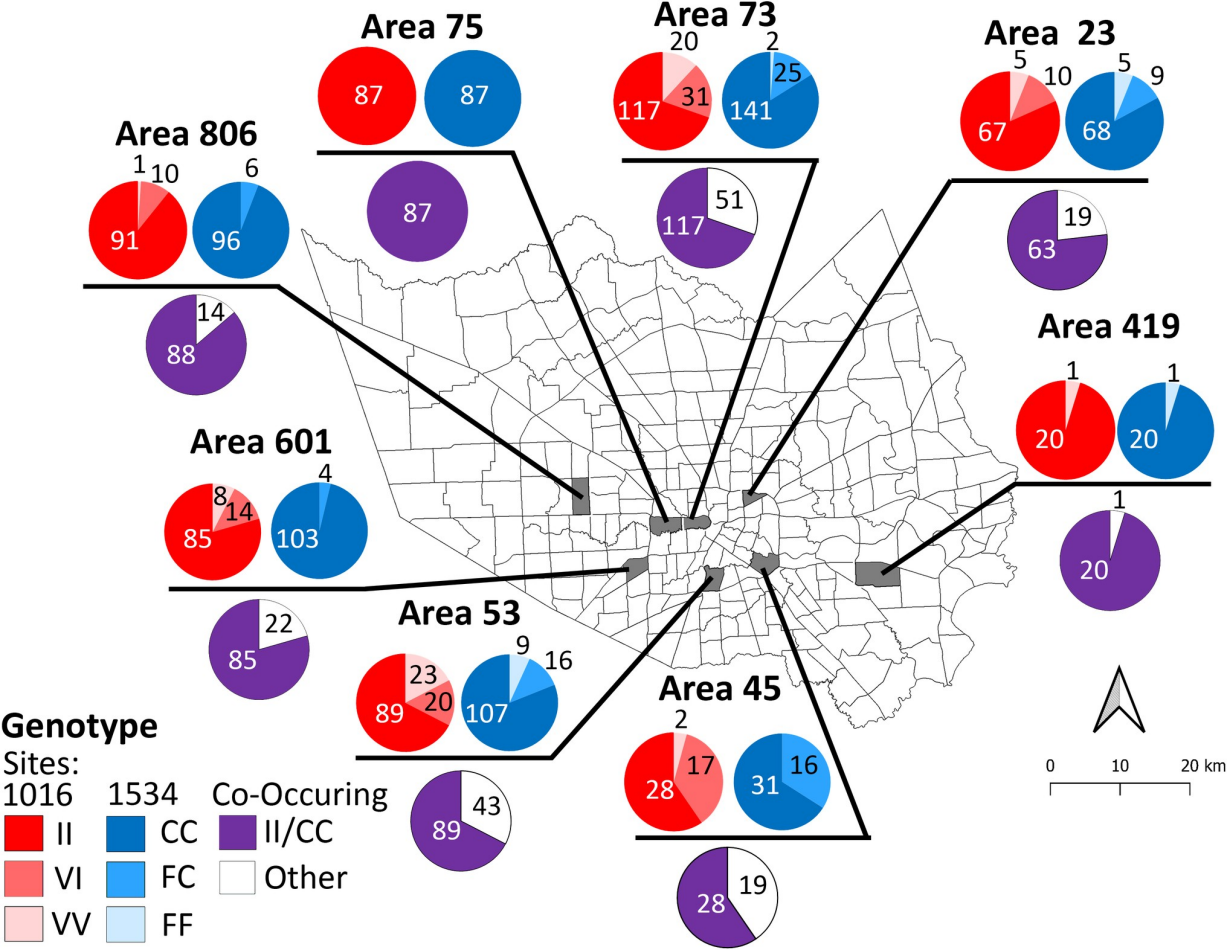
Proportion of Na_V_ channel *kdr* genotypes in Harris County. The pie charts represent the percentage of the detected *kdr* genotypes in female *Ae*. *aegypti* collected from 8 different operational areas. The proportions of genotypes for the 1016 site are shown in shades of red (II, VI, VV) and those for the 1534 site are in shades of blue (CC, FC, FF). Darkest colors indicate homozygous mutants (II in red or CC in blue), intermediate colors indicate heterozygotes (VI or FC), and the lightest color indicates susceptible genotypes (VV or FF). For each area, the proportion of double homozygous resistant (II/CC) females is shown in violet, while the proportion of all other co-occurring *kdr* genotypes at positions 1016 and 1534 (Table 2) are in white. The resulting number of genotypes per area is indicated on the corresponding color on each pie chart.

The survivorship of all areas combined was different at each distance tested (Fig 4) and the co-occurring genotypes were not equally represented in all areas (Fig 5 and Table 2). Furthermore, the proportions of II/CC genotypes among the eight different areas were statistically different (S3 Fig; Chi-square, *P* < 0.0001). A Bonferroni corrected 2-sample proportion pairwise comparison among the areas detected the highest proportion of II/CC females in areas 75 and 419 which did not differ (S3 Fig). However, areas 419 and 806 were also not significantly different from each other in the proportion of II/CC mosquitoes (S3 Fig). Areas 601, 23, 73, 53, and 45 were not significantly different from each other in the proportion of II/CC genotypes, which were less abundant than in the previously mentioned three areas (S3 Fig). While tests in earlier years (areas 419, 23, 73 and 53) detected a few double homozygous susceptible (VV/FF) genotypes (Table 2), none were found among females from the tests that were performed in 2019 (areas 75, 806, 601 and 45) (S3 Fig and Table 2). For tests performed in 2019, area 75 had 100% frequency of II/CC females, while area 45 had the lowest proportion of II/CC, at 59.6% (Figs 5 and S3 and Table 2).

When the females were analyzed for the V1016I or F1534C genotypes independently, all areas had more homozygous resistant (II or CC) individuals than either of the respective homozygous susceptible (VV or FF) or heterozygous resistant genotypes (VI or FC) (S4 Fig; Chi square, *P* < 0 .0001). The statistical analysis revealed four groups of areas for their similarity in the frequencies of the VV, VI and II genotypes, as follows: 1) areas 75, 419 and 806; 2) areas 806, 23 and 601; 3) areas 601 and 73, and 4) areas 73, 53 and 45 (S4A Fig). The latter group had more homozygous susceptible (VV) individuals and fewer homozygous resistant (II) individuals. For the V1016I site, the I1016 resistant genotype (either VI or II) was detected in 91.9% of females, being homozygous or heterozygous resistant at that site (S4A Fig and Table 2). We found that the homozygous resistant C1534 mutation (CC genotype) occurred at an overall greater frequency of 87.5% (Table 2), compared to the homozygous resistant I1016 mutation (II) at 78.2% (Table 2 and S4B Fig). For the F1534C site, the resistant genotype (either FC or CC) was present in 97.7% of females (Table 2 and S4B Fig). The statistical analysis detected three different groups: 1) Areas 75, 601, 419, and 806 genotypes; 2) areas 419, 806, 73, 23, and 53; and 3) area 45 which had the lowest occurrence of CC genotypes at 65% (S4B Fig).

Congruent with the differences found in the percentage of genotypes at each *kdr* mutation site (S4 Fig), there were significant differences in the frequency of *kdr* alleles in females across all areas (P < 0.0001) (S5 Fig). The *kdr* frequencies of the I (86.8%, S5A Fig and Table 2) and C alleles (92.6%, S5B Fig and Table 2) across the eight areas were higher than the susceptible allele frequencies (V or F, respectively) (Chi square, *P* < 0.0001 for both panels A and B). For the 1016 site, pairwise comparisons revealed four groups of areas that differ significantly: 1) Areas 75 and 419 had the highest allele I frequency (90%), followed by group 2) that included areas 419, 806, 23, and 601, 3) areas 23, 601, 73, and 45, and 4) areas 601, 73, 45, and 53 (S5A Fig). For area 53, the I allele frequency was 75% (Table 2). Area 419 could not be distinguished between groups 1 and 2; Areas 23 and 601 could not be distinguished from groups 2 and 3, and areas 601, 73, and 45 could not be distinguished from groups 3 and 4, the latter also including area 53.

For the 1534 site (F and C alleles), pairwise comparisons detected three area groups that differed in allele frequency, as follows: 1) areas 75, 601, 806, and 419, 2) areas 806, 419, and 73, and 3) areas 419, 73, 23, 53, and 45, which consequently had a higher frequency of the F susceptible allele (S5B Fig). Some areas were common among groups: area 806 could not be distinguished from groups 1 and 2, area 419 was not different from those in groups 1, 2 and 3 and area 73 could not be distinguished from groups 2 and 3. The highest frequency of C was 100% in area 75 and the lowest observed was 82.9% for area 45 (S5B Fig and Table 2).

### Impact of genotype on survivorship

**Fig 6:**
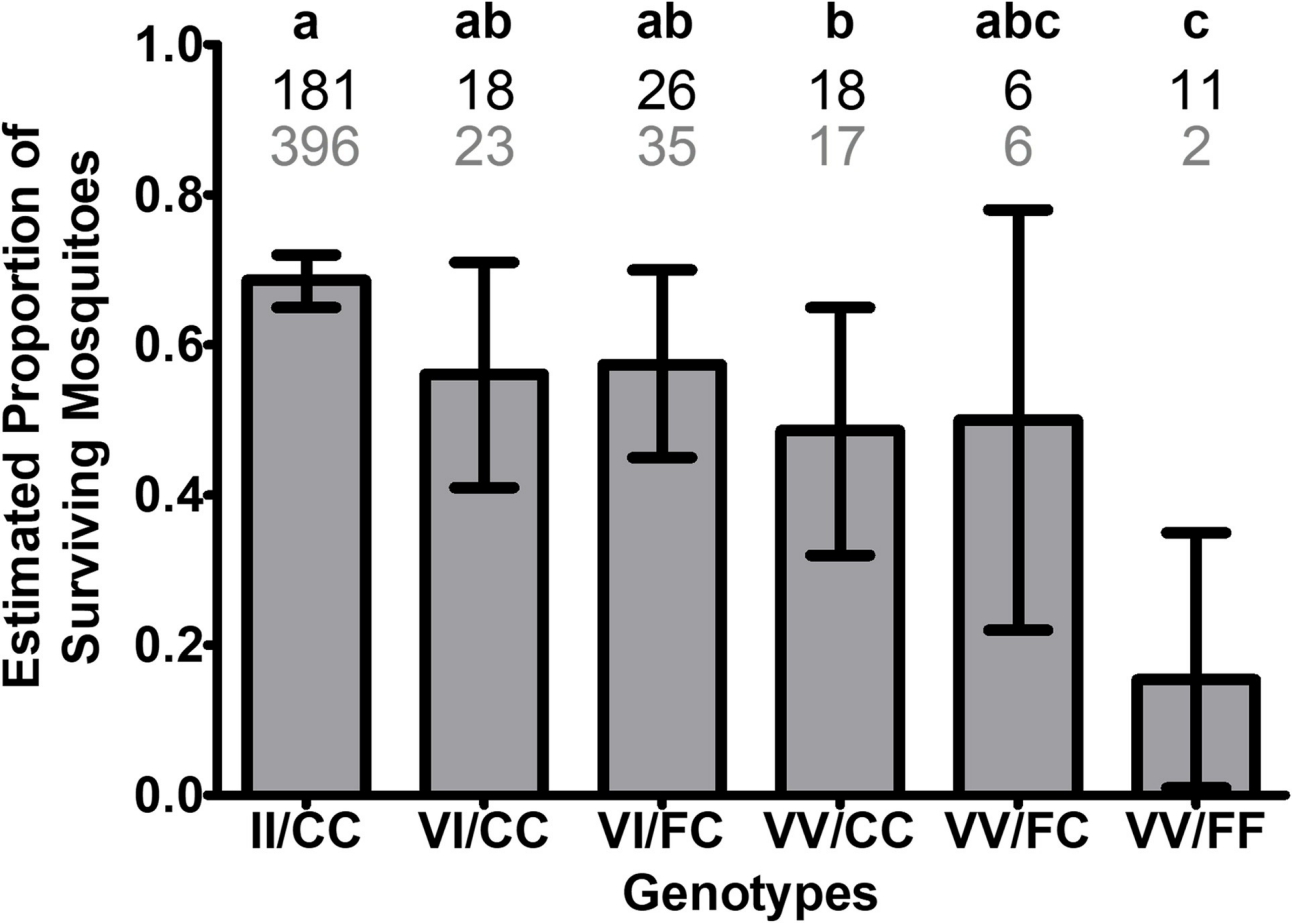
Survival status of females after Permanone 31–66 tests by co-occurring *kdr* genotypes. (A) There were significant differences in the estimated proportions of surviving *Ae*. *aegypti* females among the co-occurring genotypes at the 1016 and 1534 sites (*P* = 0.02) after adjusting for the differences among areas and distances (Cochran-Mantel-Haenszel test). The histogram shows the estimated proportions of surviving females ± CI, and different letters (a–c) above bars indicate differences in the proportions of surviving females based on paired Fisher’s Exact Tests (*P* < 0.05). Pairs that were significantly different were: (II/CC, VV/CC; *P* = 0.0241), (II/CC, VV/FF; *P* = 0.0001), (VI/CC, VV/FF; *P* = 0.0123), (VI/FC, VV/FF; *P* = 0.0124), (VV/CC, VV/FF; *P* = 0.0494). Numbers above bars indicate the number of dead (black, top row) or alive (gray, bottom row) females carrying each genotype. In addition of the number of females of respective genotypes shown in the figure, there were three females II/FC and four II/FF that were not included in the histogram or the statistical analyses, but they were included in Table 3.

We attempted to determine if the resistant genotypes (considering both mutation sites simultaneously) had an overall impact on survivorship regardless of their area of origin (Fig 6). The number of individuals with the genotypes II/FC (n = 3) and II/FF (n = 4) is considered insignificant for statistical analyses and were thus removed from analyses (Fig 6). After adjusting for differences in area and distance, there was a significant difference in the survivorship of mosquitoes among the eight genotypes recovered in this study (Cochran-Mantel-Haenszel test, *P* = 0.02) (Fig 6). For the detected genotypes at both sites 1016 and 1534, the homozygous susceptible mosquitoes (VV/FF), although relatively few (n = 13), died in their majority (n = 11; Table 3). The proportion of surviving females was the highest for the genotype II/CC (68.6%), significantly different from VV/CC that had a survival of 48.6% and from VV/FF with lowest survival of 15.4%, based on paired Fisher’s Exact test analysis among all genotypes (Fig 6). Genotypes VI/CC and VI/FC had intermediate survivorship between that of the II/CC and VV/CC genotypes of approximately 56%. The genotype VV/CC had significant higher survivorship than the susceptible VV/FF.

**Table 3:** Number of live and dead female *Aedes aegypti* in Harris County by co-occurring *kdr* genotypes detected at Na_V_ channel sites V1016I and F1534C.

Status	Co-Occurring Genotypes								
	II/CC	II/FC	II/FF	VI/CC	VI/FC	VV/CC	VV/FC	VV/FF	Total
**Dead**	181	0	1	18	26	18	6	11	272
**Live**	396	3	3	23	35	17	6	2	487

**Fig 7:**
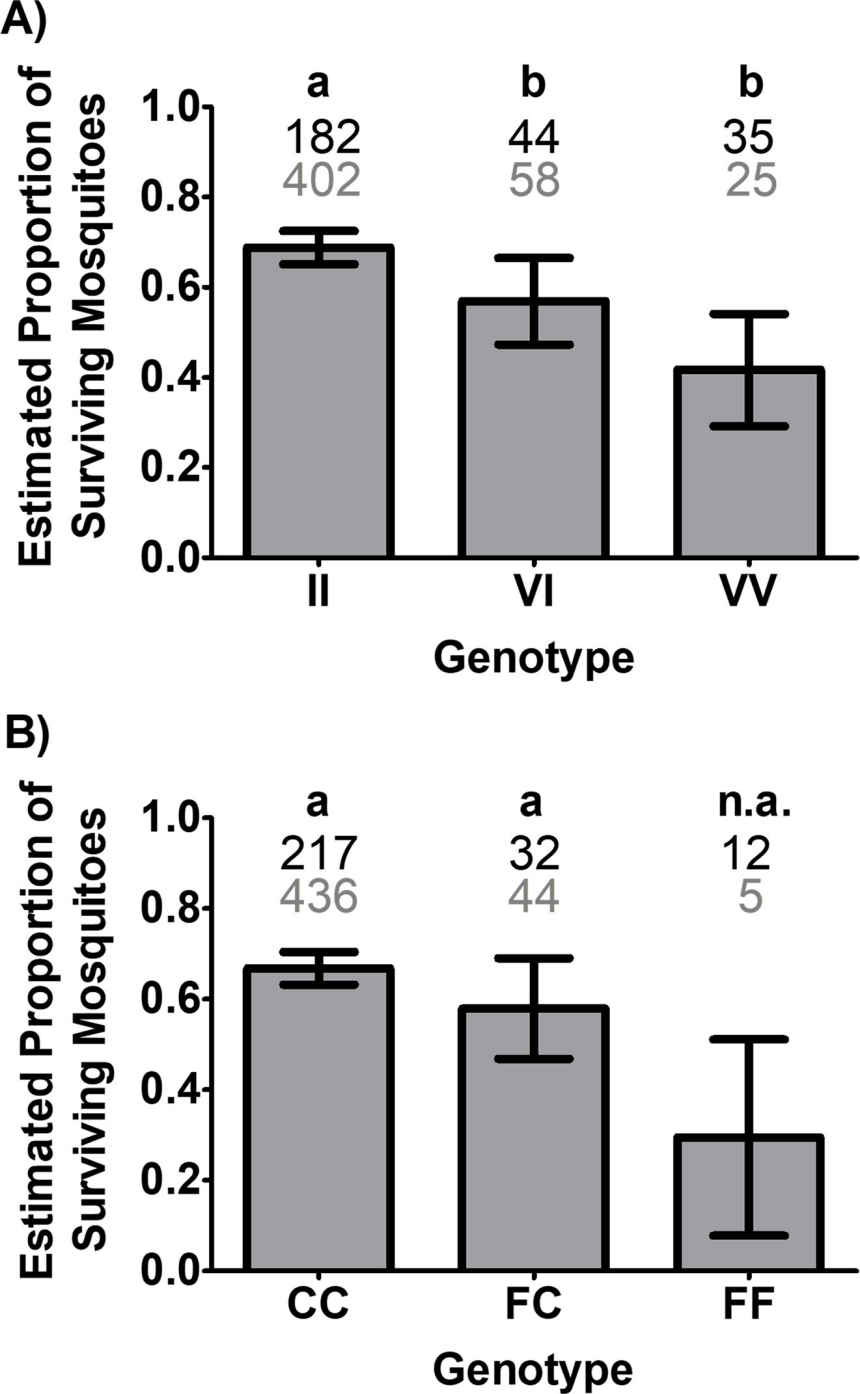
Survival status of females after Permanone 31–66 tests by *kdr* genotype at the 1016 and 1534 sites. (A) Estimated proportion of surviving females of *Ae*. *aegypti* by genotypes at the 1016 site. There were highly significant differences in the proportion of surviving females among VV, VI and II genotypes (*P* = 0.0027) after adjusting for the differences among areas and distances (Cochran-Mantel-Haenszel test). (B) Estimated proportion of surviving females of *Ae*. *aegypti* by genotype at the 1534 position. While the figure shows the survival proportion and the number of susceptible (FF) females, due to their low count, these were not included in the analyses of survivorship; differences in the proportions of live females could not be detected between the homozygous resistant (CC) and heterozygous (FC) *kdr* genotypes (Cochran-Mantel-Haenszel test; *P* = 0.4148). The histograms show the estimated proportions of surviving females ± CI, and different letters (a-b) above bars indicate differences in the proportions of surviving females (Fisher Exact Test). In A and B, numbers above bars indicate the number of dead (black, top row) or alive (gray, bottom row) females carrying each genotype.

Differences in survivorship among genotypes were then analyzed independently for each mutation site (Fig 7). For genotypes at the 1016 site, after adjusting for area and distance differences, there was a significant difference in the proportion of surviving females among the genotypes VV, VI, and II (Fig 7A; Cochran-Mantel-Haenszel test, *P* = 0.0027). The survival proportion for II was significantly higher than the survival proportions for VI and VV (Fisher Exact Test, *P* = 0.0221 and 0.0004, respectively). The survival proportions for VI and VV were not significantly different (Fisher Exact Test, *P* = 0.0740). With respect to the F1534C genotype, the proportion of surviving females across all areas and distances was not significantly different between the CC and FC genotypes (CMH, *P* = 0.4148); the FF genotype was excluded from the analysis due to low numbers detected in this study (*n* = 17; Fig 7B). These results were further supported by a Breslow-Day test of Homogeneity of the Odds Ratios across the eight areas and distances (*p* = 0.2066), indicating no significant difference in the Odds ratios of survivorship for CC and FC genotypes for all combinations of area of origin and distances. Overall, these results indicate that in Harris County, CC and FC genotypes had similar chances of survival (Fig 7B).

**Fig 8:**
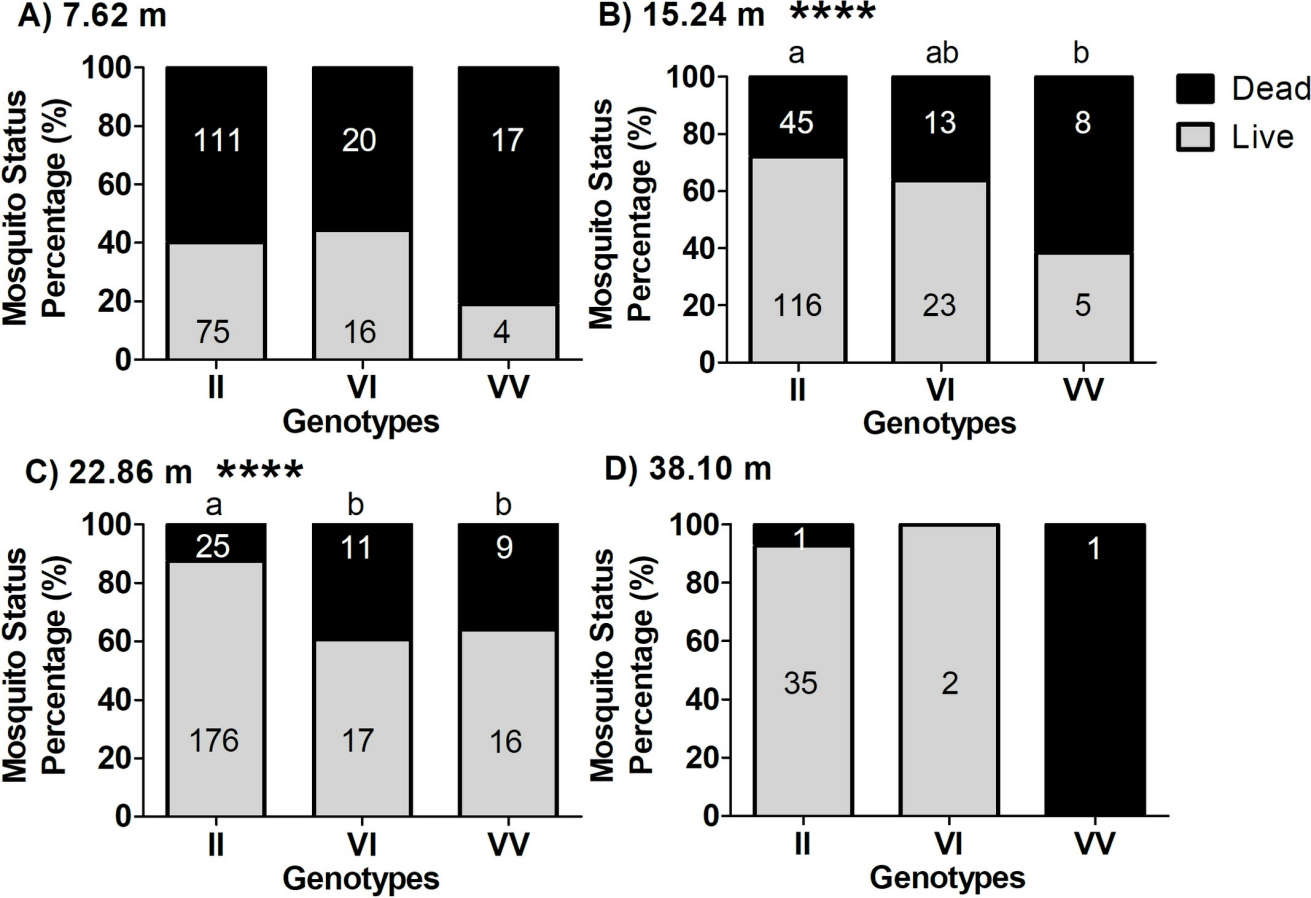
Percentage of surviving and dead females by distances and genotype at the 1016 site (V1016I). Panels show results from females of the eight areas tested at each distance from the Permanone 31–66 application source, as follows: (A) 7.62 m, (B) 15.24 m, (C) 22.86 m, and (D) 38.1 m. Numbers in black on the gray zone in each bar represent the genotyped mosquitoes that survived, and numbers in white in the black zones are the genotyped mosquitoes that died. **** indicates that at that distance there were significant differences in the proportion of surviving mosquitoes depending on their genotype (Chi-square tests: Panel B, *P* = 0.0344; Panel C, *P* < 0.0001). For panels A-C the Chi-square tests were used, and for Panel D the Fisher’s Exact test was used (*P* = 0.0526). To determine differences in survival between pairs of genotypes at both 15.24 m and 22.86 m, the Fisher’s Exact Test was performed. Results for panel 10B, at 15.24 m were: (II, VV), *P* = 0.0234; (II, VI), *P* = 0.4184; (VI, VV), *P* = 0.1902. This indicates there is a difference between the percentage of surviving females of genotypes II (homozygous *kdr*) and VV (homozygous susceptible), but there is no difference between genotypes VI and II, or VI and VV. For panel 10C, 22.86 m, results were: (II, VI), *P* = 0.0050; (II, VV), *P* = 0.0050; (VI, VV), *P* = 1.0, indicating a significant difference between the percentage of surviving females between II versus VI or VV, but no difference between VI and VV.

**Fig 9:**
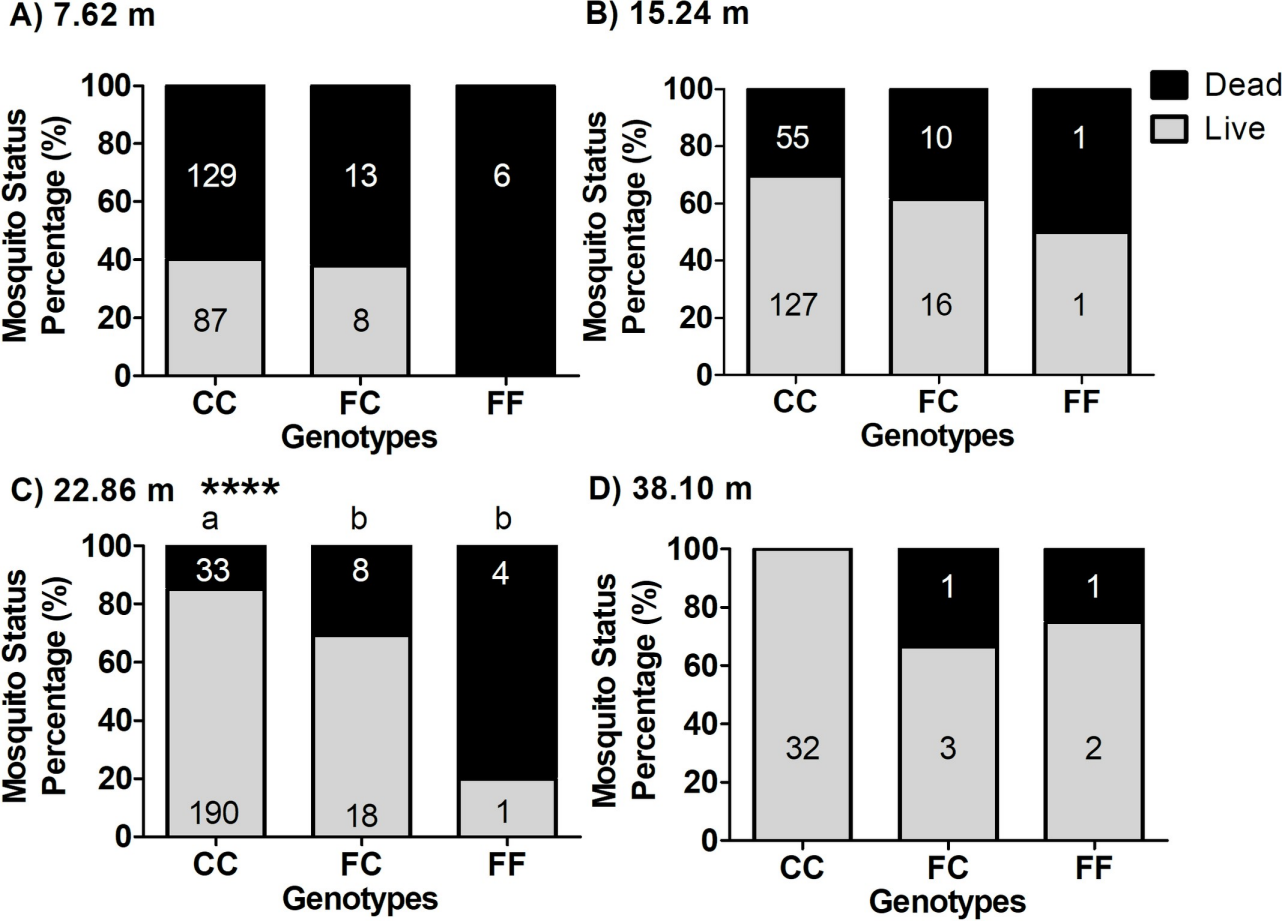
Percentage of surviving or dead females by distances and genotype at the 1534 site (F1534C). Panels show results from females of the eight areas tested at each distance from the Permanone 31–66 application source, as follows: (A) 7.62 m, (B) 15.24 m, (C) 22.86 m, and (D) 38.1 m. Numbers in black on the gray zone in each bar represent the genotyped mosquitoes that survived, and numbers in white in the black zones are the genotyped mosquitoes that perished. **** indicate at that distance, there were significant differences in the proportion of surviving mosquitoes depending on their genotype (Fisher’s exact test: Panel C, *P* = 0.0005). To determine differences in survival between pairs of genotypes at 22.86 m, the Fisher’s Exact Test was performed, and results were: (CC, FC), *P* = 0.0497; (CC, FF), *P* = 0.0027; (FC, FF), *P* = 0.06.

Survivorship results of co-occurring genotypes at each of the distance tested could not be statistically analyzed due to the lack of representation of genotypes at the different distances (S6 Fig). We then analyzed the genotype effect on survival at each distance, independently for the 1016 site (Fig 8) and the 1534 site (Fig 9), for all areas combined. For the 1016 site, there were no differences in survivorship among genotypes at the shorter distance of 7.62 m (Fig 8A; Chi-square, *P* = 0.1291) from the Permanone 31–66 application. The genotypes differed in survivorship in the trials at 15.24 m (Fig 8B, Chi-square, *P* = 0.0344) and at 22.86 m (Fig 8C, Chi-square, *P* < 0.0001). At 38.1 m, only one susceptible female VV died from the application, thus, the test did not detect significant differences between the survivorship of the resistant (II) and susceptible (VV) genotypes (Fig 8D), although the *P* value of the Fisher Exact Test (*P* = 0.0526), was close to detect significance.

For the 1534 mutation site, there were no significant differences in survival among the observed genotypes at 7.62 m (Fig 9A; Fisher Exact Test, *P* = 0.162) and at 15.24 m (Fig 9B; Fisher Exact Test, *P* = 0.699). However, there was a significant difference in survivorship among the F1534C genotypes CC, FC, and FF at 22.86 m (Fig 9C; Fisher Exact Test *P* = 0.0005), with the pairs (CC, FC) and (CC, FF) having significantly different survival proportions but not the pair (FC, FF). At 38.1 m, there was not a significant difference in survivorship among any of the pairs of the F1534C genotypes (CC, FC; *P* = 0.0857), (CC, FF; *P* = 1.0), and (FF, FC; *P* = 0.1111) (Fig 9D; *P* refers to Fisher Exact Test).

### Impact of factors influencing survival using a logistic regression model

For the analyses using a logistic regression model, the genotypic data of females from 38.1 m [area 23; n = 39] were removed from the analysis because only for area 23 were females tested at that distance. Models for V1016I (S7A Fig) and F1534C (S7B Fig) were fit to the data with interaction terms, but none were significant, so a main effects model was then fit to the data yielding the results. The logistic regression fitted models indicated there is a significant difference in the predicted probabilities of survival across the 24 combinations of area and genotype with 95% CIs (S7 Fig). The graphs show the probability of survival for each genotype and area, but it is not to be interpreted to contrast probability of survival between specific areas and genotypes.

At the 1016 position, the analysis identified area (*P* < 0 .0001), distance (*P* < 0.0001), and cage within each distance (*P* < 0.0001) as significant effects on survivorship. Genotype at the 1016 position was not shown to be significant on survival (*P* = 0.2067) (S7A Fig). The linear model produced estimates of the Odds Ratios of survival. The Odds Ratio between the 1016 site genotypes for II vs VI is 1.67 with a 95% Confidence Interval (CI) (0.77, 3.65); II vs VV is 1.88 with a 95% CI (0.78, 4.55); VI vs VV is 1.12 with a 95% CI (0.38, 2.33). Because 1 is contained in the interval for all comparisons, the odds of survival among the genotypes are not significantly different at the 1016 site.

A second model was fit for the 1534 mutation site, and the model identified area (*P* < 0.0001), distance (*P* < 0 .0001), and cage within each distance (*P* < 0 .0001) as significant effects on survivorship, while genotype did not show significance (*P* = 0.21) (S7B Fig). From the fitted model, the Odds Ratios of survival between the 1534 site genotypes are: for CC vs FC is 1.21with a 95% CI (0.53, 2.76); CC vs FF is 5.58 with a 95% CI (0.78, 39.71); and for FC vs FF is 4.61 with a 95% CI (0.57, 3726). Due to 1 being contained in the confidence intervals for all genotype comparisons (CC vs FC; CC vs FF; FC vs FF) the odds of survival are not significantly different from each other among those genotype survival comparisons.

The high variability in the number of mosquitoes for each area and genotype (Table 2) further precludes from pairwise comparisons of the 24 combinations of area and genotype. For the 1016 genotypes, the probability of survival tends to increase in genotypes with more resistant I alleles (S7A Fig). At the 1534 position, while statistical differences could not be detected in the probability of survival among genotypes in the different areas, there is a trend for the survivorship of CC and FC to be higher than that of FF, with probability of survival for FF being the most variable due in part to the low counts for FF (n = 17) (S7B Fig). However, for both mutation sites 1016 and 1534, these differences are not significantly different (S7 Fig).

**Fig 10:**
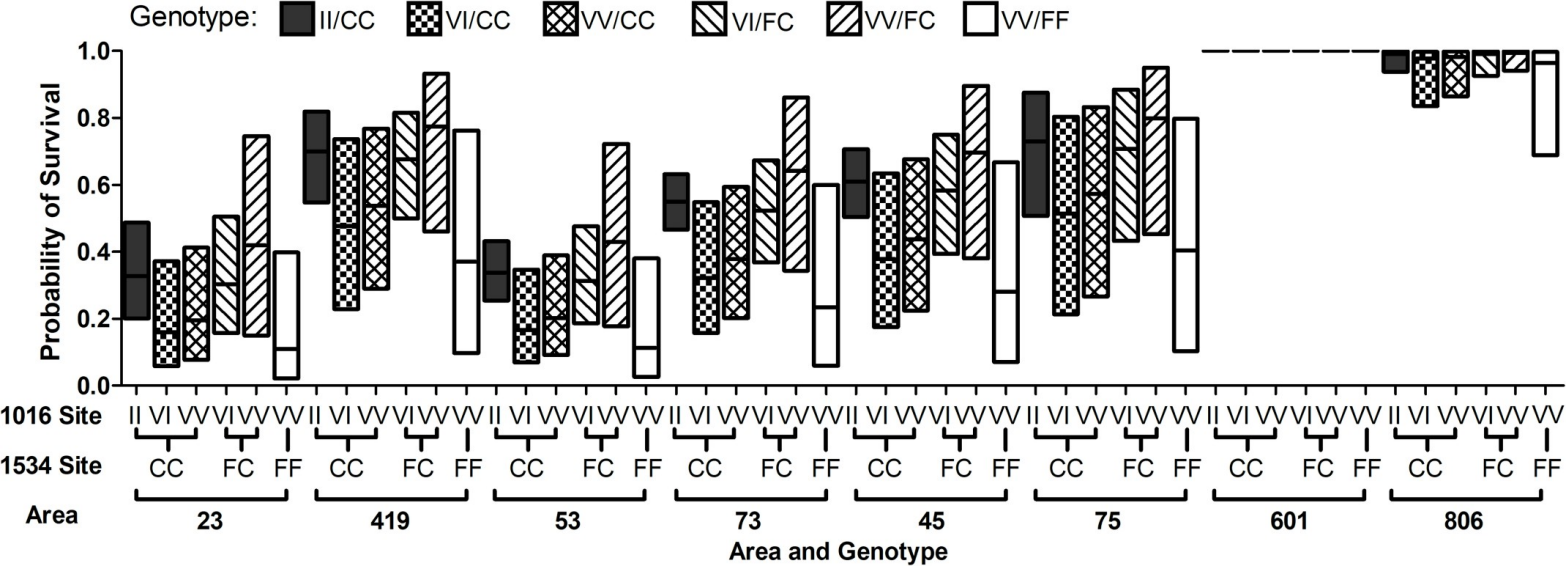
Survival probability of *Ae*. *aegypti* by area and co-occurring genotypes at sites 1016 and 1534. Probability of survival of females of *Ae*. *aegypti* with different co-occurring genotypes at the 1016 and 1534 sites. The boxes’ middle lines represent the predicted probability of survival obtained by logistic regression analysis. The top and bottom values of the boxes are the upper and lower 95% CIs. The overall model fit (logistic regression, P < 0.0001). The logistic model detected area, distance and cage influencing survivorship, but not genotype. An asymptomatic Wald’s procedure was used to obtain the CIs for the probability of survival.

A logistic regression model could not be fit for the probability of survival for area and genotype when all genotypic data for both mutation sites were used (females n = 746), because the model was affected by the low counts of some of the co-occurring *kdr* genotypes present in females analyzed. To improve the model, these genotypes II/FC (n = 3) and II/FF (n = 4) were eliminated. The logistic regression model was then fit for the co-occurring *kdr* genotypes at 1016 and 1534 sites and produced survival probabilities for areas and genotypes (Fig 10). The analyses identified area (*P* < 0 .0001), distance (*P* < 0.0001), and cage within each distance (*P* < 0.0001) as having significant effects on survivorship (Fig 10). Genotype at both positions, 1016 and 1534, did not have a significant effect on survival (*P* = 0.275) (Fig 10). Areas 601 and 806 had an overall higher probability of survivorship compared to all other areas, regardless of genotype (Figs 10 and S7).

## Discussion

### Field cage assays

To assess the impact of *kdr* mutations on the prospective operational control of *Ae*. *aegypti*, we first determined the in-field efficacy of the pyrethroid-based Permanone 31–66 applied against females from eight operational areas from Harris County. These field tests simulated a real control scenario by applying Permanone 31–66 towards mosquito cages containing females, placed at three different distances from the hand-held sprayers (Fig 2). The survivors and dead females from these trials (Figs 3 and S2) were genotyped for the V1016I and F1534C *kdr* mutations (Fig 5). Field tests of this magnitude are not commonly performed for mosquito insecticide resistance surveillance, and fewer field tests use molecular analyses to determine the impact of target site insensitivity at the Na_V_ channel on vector control. Other studies that have investigated both resistance mechanisms and in-field resistance to insecticides have performed these tests independently, which limits the understanding on how each resistance genotype may impact survival of females in the field when considering other factors such as distance [41–43].

In this study, field bioassays were performed in different dates and years, and despite these sources of variation, the Orlando strain susceptible females died consistently. Conversely, females from all Harris County areas investigated showed high variation in survivorship (Fig 3), and increasingly survived as distance from the Permanone 31–66 application increased (Figs 3 and 4). These results are supported by previous work in which strain and distance from the spray source were key factors associated with mortality rates in *Ae*. *aegypti* [43,44].

Survivorship was higher than expected considering the current lack of targeted public control for *Ae*. *aegypti*, even at the shortest distance of 7.62 m with ~40% survival (Figs 3 and 4). Regardless, most mosquitoes did die at 7.62 m from the Permanone 31–66 application (Figs 3, 4, and S2), suggesting a relatively higher and/or homogeneous distribution of the applied insecticide at this distance. Noted exceptions were all females from areas 601 and 806, which had 100% survival even at 7.62 m (Figs 3A and S2A).

The percent mortality at short distance in Harris County contrasts with results of field assays performed in Florida where mid-rate applications of permethrin had 64% survivorship of females at 7.6 m and 15.2 m from the spray source [45]. The higher survivorship in field assays in Florida vs. Harris County might be explained by the fact that assays in Harris County tested the highest recommended rate of application. Several factors affect mortality results in field tests as evidenced by a variable survivorship of Orlando females at different distances (S2 Fig). Among such factors is the type of fogger used. In a similar study with susceptible *Ae*. *aegypti* Rockefeller strain and comparing applications of permethrin + pyriproxyfen, the cold fogging resulted in 95% mortality at 6 m and about 90% at 9 m, while thermal fogging resulted in 100% mortality. The output of a cold fogger, such as the one used here, provides excellent droplet size but does not penetrate obstacles (i.e., cages) as the thermal fogging does [37]. Wind direction and speed and local thermal inversion also influence the spread of the insecticide application. Field variability in application in Harris County tests is supported by the logistic regression model, which found significant differences in survivorship among areas, distance, and cages within each distance, but not genotypes (Fig 10). Additionally, outdoor conditions may have affected effectiveness of Permanone 31–66 to control *Ae*. *aegypti* females at 22.86 m, supporting the fogger manufacturer’s recommendation of treating outdoor areas at 15.24 m increments [27]. All these factors may explain the survival of susceptible Orlando mosquitoes at different distances in the field tests for areas 73, 45, 75, 601, and 806 (S2 Fig). With a 25% probability of failed control (assays on Nov. 6, 2018, and Aug. 6, 2019, out of eight performed; S2 Fig), this work exemplifies the challenge of controlling *Ae*. *aegypti* in real situations.

### Genotype variation in Harris County areas

The high survivorship in field bioassays in Harris County prompted investigation into the potential presence of *kdr* mutations F1534C and V1016I, which had previously been detected in North American populations of *Ae*. *aegypti* from Arizona, California, Florida, Louisiana, and New Mexico [20,46–48]. Indeed, high frequencies of *kdr* alleles resulted from the field collected samples, as 98.3% of all tested mosquitoes had at least one resistant allele (Fig 5 and Table 2). These *kdr* analyses are the first report of *kdr* mutations in *Ae*. *aegypti* from Harris County, TX, where a population of 4.7 million people may be at risk for its transmitted arboviruses.

The large numbers of double homozygous resistant (II/CC) mosquitoes across all study areas likely contributed to the high female survivorship at 15.2 and 22. 86 m (Table 2 and Figs 3 and 4). Previous studies detected high proportions of *kdr* mutations in populations of this species subjected to high frequency of permethrin use, including those from Mexico, Florida, and California [41,46,49]. In agreement with other reports [46,50], in Harris County the II/CC genotype conferred higher pyrethroid survivorship than the susceptible VV/FF genotypes (Fig 6). As the V1016I/F1534C double mutants have increased resistance to type I and type II pyrethroids, operational areas with high frequency of II/CC mosquitoes (Figs 7 and S6) would be limited in alternative control tactics as populations will be cross resistant to deltamethrin-based formulations [22].

The II genotype at site 1016 is proposed to have low fitness and to have evolved after the F1534C mutation, which may explain the low abundance in Harris County of II/FF females (n = 4; 0.5%), only found in area 23, and of II/FC females (n = 3), only found in area 806 [23,25]. The I allele has been associated with early mortality in eggs and larvae in the absence of pyrethroid exposure [51,52]. The low occurrence of the II/FF genotype was reported in other studies which found it at even lower percentages than in Harris County, from 0.05 to 0.1% [25,46]. In addition to the selection by Permanone 31–66 in area 23, the detection of this rare genotype in Harris County could be due to at least two reasons. First, our intensive sampling within each operational area where collections were done in eight different locations. Second, that in this study adults of the F_0_ generation were used for genotyping, reflecting the genetic diversity existing in the field. Other studies genotyped F_1_ or F_2_ adults and in doing so, the rare II/FF and II/FC field-collected genotypes could have been lost during laboratory rearing [25,46].

No susceptible females (VV/FF) were recovered from areas 45, 75, 601, and 806 in 2019 (Table 2 and S3 Fig), which may have contributed to these areas having high survivorship rates (S2B Fig). The high survivorship at 7.62 m of females in areas 601 and 806 (Fig 3) could be explained by their high percentage of double-homozygous resistant females (II/CC), 79.4% and 86.3% respectively (Table 2); the absence of detected susceptible mosquitoes, and their high proportion of CC genotypes (S4B Fig) and consequently, a very high frequency for the C allele (S5B Fig).

Area 75 has 40% more double homozygous *kdr* resistant mosquitoes than area 45 (Fig 5), these areas are about 16 km apart within Interloop 610 in Houston, and their field tests were conducted on the same day. Pyrethroid selection pressure was higher for area 75 where the HCPH-MVCD applied three Permanone 31–66 treatments in 2019 before the field-cage test was performed. Area 75 includes a high-income residential area (with property values $400,000- $1.2 million), Memorial Park, and the Houston Arboretum. Area 45 includes the Buffalo Bayou on its northern border and is primarily a residential area with lower property values, ranging from $250,000-$450,000. Thus, it is also likely that more affluent residents in area 75 may contract private mosquito control services [28], which may increase the pyrethroid selection pressure for *kdr* mutations.

### Influence of genotype on survival

The independent statistical analyses for each mutation site genotype (Fig 7), performed because of the low numbers for some co-occurring genotypes (II/FC, II/FF, VV/FF) (S6 Fig and Table 2), estimated that females with II at the 1016 site were more likely to survive exposure to Permanone 31–66 than VV and VI females, while analyses for the 1534 genotype instead estimated similar survival for both the CC and FC genotypes (Fig 7). As the 1016 and 1534 sites are approximately 44.5 kb apart, these sites are linked and both influence female survivorship, as shown in previous studies [25,53].

When considering distance and genotype for both mutation sites simultaneously (S6 Fig), the genotype of females at 7.62 m did not have an impact on survival, suggesting that at close distances higher probability of exposure to pyrethroids will result in mortality regardless of resistant genotype (Figs 8, 9, and S6). At 38.1 m only two females died as the amount of pesticide received was likely too low and the fog concentration variable. In addition, and serendipitously, most mosquitoes at that distance resulted double homozygous resistant, all which may have resulted in high survival (S6D Fig). The resistant genotypes influenced survivorship at 15.24 and 22.86 m away from the spray source (Figs 8 and 9). Higher survivorship of CC at 22.86 m is important because at these longer distances, the sprayers deliver less homogeneous coverage. While it is reasonable to conclude that the CC and FC females would have had an advantage at 15.24 m over the susceptible FF, we could not statistically detect these differences because only two had the FF genotype (Fig 9B). In summary, genotype significantly influenced survival at 22.86 m for both mutation sites (Figs 8C and 9C).

### Joint analyses of factors influencing survival

The combination of the wide distribution and high frequency of resistant genotypes and high survivorship when exposed to Permanone 31–66 may pose a challenge for vector control. For these reasons, we tested a linear model to determine the probability of survival based on genotype at each *kdr* site, area, and cage within the same distance in the field test (S7 Fig). When all factors were considered, the logistic regression model did not find statistical differences in the probability of survival for any of the genotypes analyzed independently for the 1016 and 1534 sites. For each mutation site (S7A and S7B Fig) there are differences in the predicted probability of survival across twenty-four combinations of areas and genotypes. In the analysis of the probability of survival for the 1016 site, although the figure shows a trend suggesting that resistant alleles (I) at the 1016 position (S7A Fig) increase resistance in an additive manner, the analyses of odds ratios of survival failed to identify differences among the three genotypes. For the resistant allele at position 1534, the logistic regression model did not find significant differences in probability of survival when all factors were considered, the odds ratio analysis also failed to demonstrate differences in survivorship between the three genotypes. While the *kdr* F1534C mutation is considered to be incompletely recessive [54], our independent analysis of mutation sites found F1534C apparently dominant (Fig 7). However, the linear model did not detect statistically significant differences in the survival probability of the CC vs FC genotypes but detected significant effects of area, distance, and cages within each distance.

Different from other studies, the model showed a trend for dominance of the C allele at site 1534 (similar probability of survival for CC and FC genotypes) for all areas (S7B Fig). It has been recently established that the *kdr* mutation at the 1534 site provides relatively low resistance ratios values, ranging from 7–16 [22]. Heterozygous genotypes at other sites in addition to the *kdr* 1534 site provide increased resistance to pyrethroid treatment [55]. Our data show that 84% of females carrying the FC apparently dominant genotype (S7 Fig) also carry at least one I allele (Fig 10 and Table 2) at position 1016 (Figs 6 and S6), or two II alleles (Fig 6), which likely contribute to higher resistance ratios in the field.

The application rate in Harris County is the highest allowable for Permanone 31–66, but the treatment efficacy varied by distance. Our analyses showed that for *kdr* at the 1534 site, mortality at shorter distances (less than 22.6 m) was still high and similar for both CC and FC genotypes because of the highest concentration of pesticide received conveying apparent dominance to the C mutation (Fig 9A and 9B). Only at the distance of 22.36 m the CC and FC genotypes have differential mortality (Fig 9C). However, if the *kdr* genotype at 1534 is not accompanied by the I mutations at the 1016 site, it appears that mortality at 22.6 m is more random for CC and FC (higher observed mortality of VV/CC vs. lower for VV/FC, contrary to expectations, S6C Fig) because the application of insecticide at that distance is likely less uniform. Likely, variations in the insecticide fog prevented differentiating FC from CC genotypes in the absence of any I allele because *kdr* at 1534 only provides relatively low levels of resistance. In our bioassays, serendipitously, the group of mosquitoes placed in the cages at 22.86 m also resulted in the highest number of VV/CC genotypes among the different distances tested (S6 Fig), fact that could have helped in evaluating the effect on survivorship of the *kdr* mutation at 1534 (CC or FC) independently of the presence of the I allele (i.e., VI and II genotypes) (Fig 9). In sum, the presence of VI in combination with FC genotype may tend to increase probability of survival with respect to the VI/CC genotype although this difference is not statistically detected by the model (Fig 10).

The potential presence of other *kdr* mutations on the Na_v_ channel not analyzed here, such as V410L, may also contribute to the high survivorship in Harris County populations [56]. While *kdr* mutations synergistically provide higher levels of resistance to pyrethroids than metabolic resistance, these two different mechanisms can act in combination to grant greatly increased resistance to different pyrethroids [57]. Cytochrome P450 monooxygenases could potentially contribute to *Ae*. *aegypti* resistance to pyrethroids [58], but they were not analyzed here. Permanone 31–66 contains their inhibitor PBO, likely reducing their contribution, if any, to *Aedes* survival in these bioassays.

## Conclusion

This study shows that permethrin resistance is present in *Ae*. *aegypti* throughout Harris County and most female mosquitoes analyzed had both *kdr* mutations. Despite this species not being the intended target of the HCPH-MVCD pyrethroid applications, resistance has developed within the *Ae*. *aegypti* population of Harris County. In Texas, the only previous report of pyrethroid resistance in *Ae*. *aegypti* was in 2018, detected by bottle bioassays conducted in Dallas, but the resistance mechanism was not investigated [59]. As for the origin of the *kdr* mutations, local selection pressure by pyrethroid applications appears to be associated with operational area differences in pyrethroid resistance, as applications ranged from 0 to 16 per area and year, from 2011–2019, with higher number of treatments in all areas analyzed from 2012–2014. The population genetic structure of *Ae*. *aegypti* from Houston, TX, includes markers from the Southwestern USA as well as the Southeast USA [60]. The potential current invasion of mosquitoes from the Southwestern USA into Harris County appears less likely as Kandel et al. (2019) reported that only the F1534C mutation was found in females from six cities in Southern New Mexico out of six mutations investigated. Invasions from New Orleans populations could be more likely as both the F1534C and V1016I were reported in those [20].

Harris County may be at a higher risk of arboviral transmission from *Ae*. *aegypti* if insecticide applications are not delivered at relatively short distances when arboviruses are detected, at about 7.62 meters and up to 15.24 m. The results show that resistance is not homogenous across Harris County and that more testing in additional operational areas is needed to assess the resistance status throughout to improve control. This is the most complete study on USA populations of *Ae*. *aegypti* mosquitoes for an urban area simultaneously assessing mortality in field bioassays, *kdr* genotyping and evaluating multiple factors on their direct effects on vector control.

## Supporting information

S1 Fig Materials used for field collection of eggs and insectary rearing of *Aedes aegypti*. (A) Ovicups used to collect *Aedes* eggs in the field. (B) Paper used for oviposition, showing *Aedes* eggs (black masses). (C) Cages with sugar feeders used for rearing field and laboratory (Orlando) strains of *Ae*. *aegypti*. (D) Blood-feeder on top of cages containing sheep blood kept at 37°C for feeding *Aedes* mosquitoes. (E) Top view of a water-filled steel pan with *Aedes* eggs-covered paper used for larval rearing.
(TIF)

S2 Fig Survivorship percentage of *Ae*. *aegypti* at different distances in comparison to control Orlando females. Permanone 31–66 was applied at (A) 7.62 m from cages; (B) 15.24 m; (C) at 22.86 m; or (D) 38.1 m. Each bar represents the mean ± SD of 2–3 cages per distance. There were significant differences at all distances in the average survivorship between the field collected females and the Orlando strain females tested in the treatment zone (Chi-Square Test; *P* < 0.0001). The analyses were followed by pairwise comparison tests with Bonferroni correction to detect significant differences between the field mosquitoes and their treated Orlando controls. Asterisks above bars indicate the areas with significantly different survivorship (*P* < 0.05) from the Orlando (Or) females. For each area, histograms show Mean ± SD.
(TIF)

S3 Fig Percentage of *Ae*. *aegypti* double *kdr* genotypes II (1016)/CC (1534) vs. other genotypes, per area. The numbers in the graph refer to the number of mosquitoes analyzed for each area: in black above each bar are the number of susceptible (VV/FF) mosquitoes; white numbers on black bars are double homozygous resistant (II/CC) females; in black on grey areas are the number of mosquitoes of all other genotypes that were not VV/FF or II/CC. Horizontal lines above bars indicate the areas which are not significantly different (*P* < 0.05).
(TIF)

S4 Fig Percentage of genotypes detected per area for each mutation site, V1016I and F1534C, shown independently. Percentage of the (A) V1016I mutation and (B) F1534C mutation in females of *Ae aegypti*. Significant differences were detected among the proportion of genotypes observed across all areas for each mutation site analyzed independently (Chi-square; *P* < 0.0001 for each, A and B panels). Horizontal lines above bars indicate areas in which the percentage of genotypes are not significantly different (*P* < 0.05). Numbers above bars indicate the number of susceptible field-collected mosquitoes (either VV or FF, respectively) detected. Numbers in white on black bars are double homozygous resistant (II or CC) females; in white on grey areas are the number of heterozygous females (VI or FC, respectively).
(TIF)

S5 Fig Percentage of *kdr* alleles detected in *Ae*. *aegypti* from each area. Percentage of the (A) V and I alleles at position 1016, and (B) F and C alleles at position 1534. Significant differences were detected across all areas between the proportion of the two alleles V and I (A), or F and C (B) for each mutation site (Chi-square; *P* < 0.0001 for each panel). Horizontal lines above bars indicate the areas which are not significantly different (*P* < 0.05). In each panel, note that areas are organized in order of decreasing frequency of the resistant allele. Note that areas are grouped differently for their similarity in the frequency of each of the resistant alleles.
(TIF)

S6 Fig Percentage of surviving and dead females at different distances by co-occurring genotype at 1016 and 1534 sites. Panels show results of each of the tested distances from the Permanone 31–66 application source, as follows: (A) 7.62 m, (B) 15.24 m, (C) 22.86, and (D) 38.1 m. In black on the gray zone in each bar is the number of genotyped mosquitoes that survived, and in white in the black zone are the number of genotyped mosquitoes that perished. NF: none found. The data as shown here could not be statistically analyzed due to the lack of individuals for some genotypes in different areas and for some distances. This figure supplements data in Fig 6 in the main text.
(TIF)

S7 Fig Survival probability of *Ae*. *aegypti* by area and genotype (V1016I or F1534C) shown independently. Probability of survival of females of *Ae*. *aegypti* carrying the (A) V1016I mutation or (B) the F1534C mutation. The boxes’ middle lines represent the predicted probability of survival obtained by logistic regression analysis. The top and bottom values of the boxes are the upper and lower 95% CIs. The overall model fit (logistic regression, P < 0.0001). An asymptomatic Wald’s procedure was used to obtain the CIs for the probability of survival.
(TIF)
