# Supplementary figures and images for: Kdr genotyping (V1016I, F1534C) of the Nav channel of Aedes aegypti (L.) mosquito populations in Harris County (Houston), Texas, USA, after Permanone 31–66 field tests and its influence on probability of survival
Source: PLoS Negl Trop Dis. 2021 Nov 4;15(11):e0009833. doi: 10.1371/journal.pntd.0009833 (PMC8568202; doi:10.1371/journal.pntd.0009833)

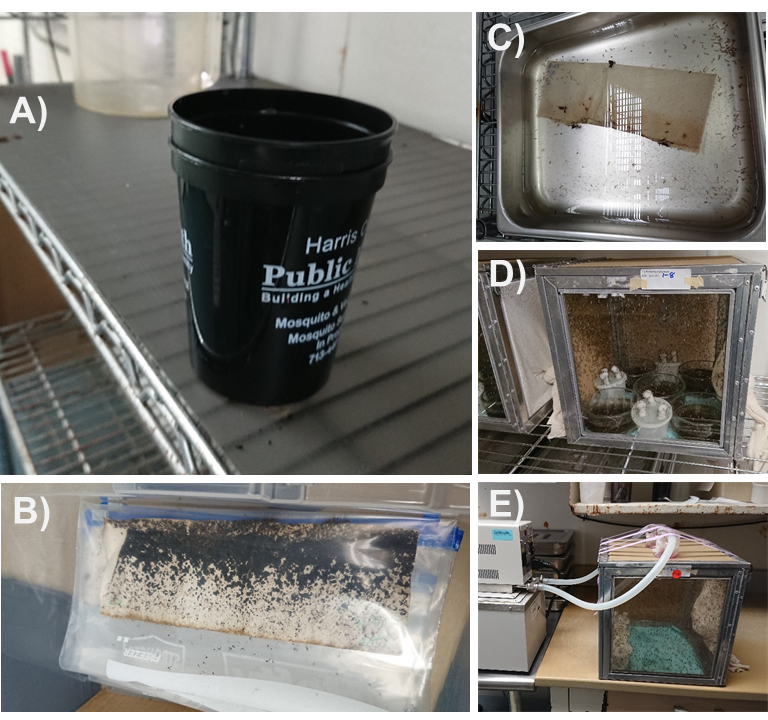

Supplement: S1 Fig — (A) Ovicups used to collect Aedes eggs in the field. (B) Paper used for oviposition, showing Aedes eggs (black masses). (C) Cages with sugar feeders used for rearing field and laboratory (Orlando) strains of Ae. aegypti. (D) Blood-feeder on top of cages containing sheep blood kept at 37°C for feeding Aedes mosquitoes. (E) Top view of a water-filled steel pan with Aedes eggs-covered paper used for larval rearing. (TIF) [file pntd.0009833.s001.tif]

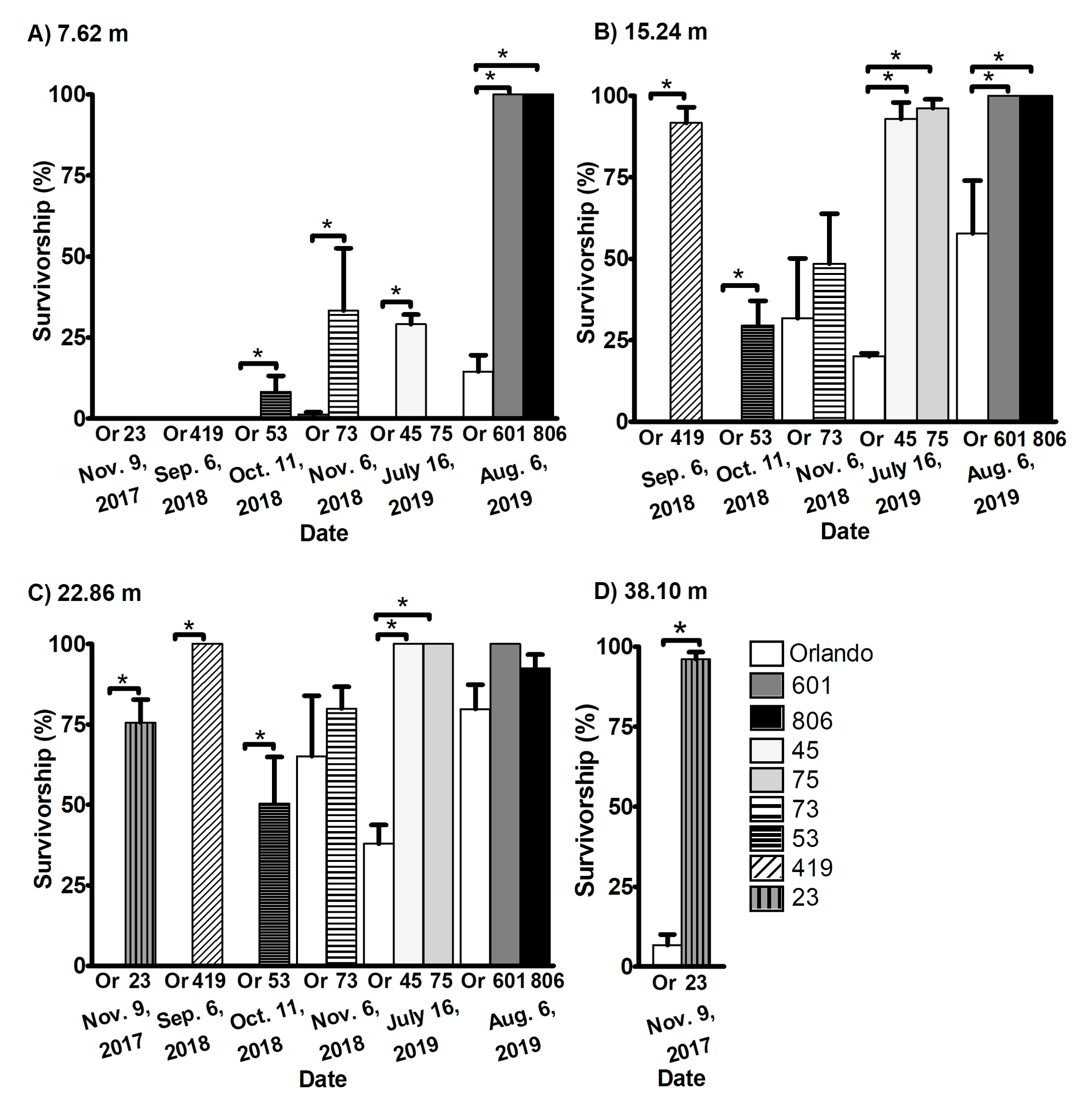

Supplement: S2 Fig — Permanone 31–66 was applied at (A) 7.62 m from cages; (B) 15.24 m; (C) at 22.86 m; or (D) 38.1 m. Each bar represents the mean ± SD of 2–3 cages per distance. There were significant differences at all distances in the average survivorship between the field collected females and the Orlando strain females tested in the treatment zone (Chi-Square Test; P < 0.0001). The analyses were followed by pairwise comparison tests with Bonferroni correction to detect significant differences between the field mosquitoes and their treated Orlando controls. Asterisks above bars indicate the areas with significantly different survivorship (P < 0.05) from the Orlando (Or) females. For each area, histograms show Mean ± SD. (TIF) [file pntd.0009833.s002.tif]

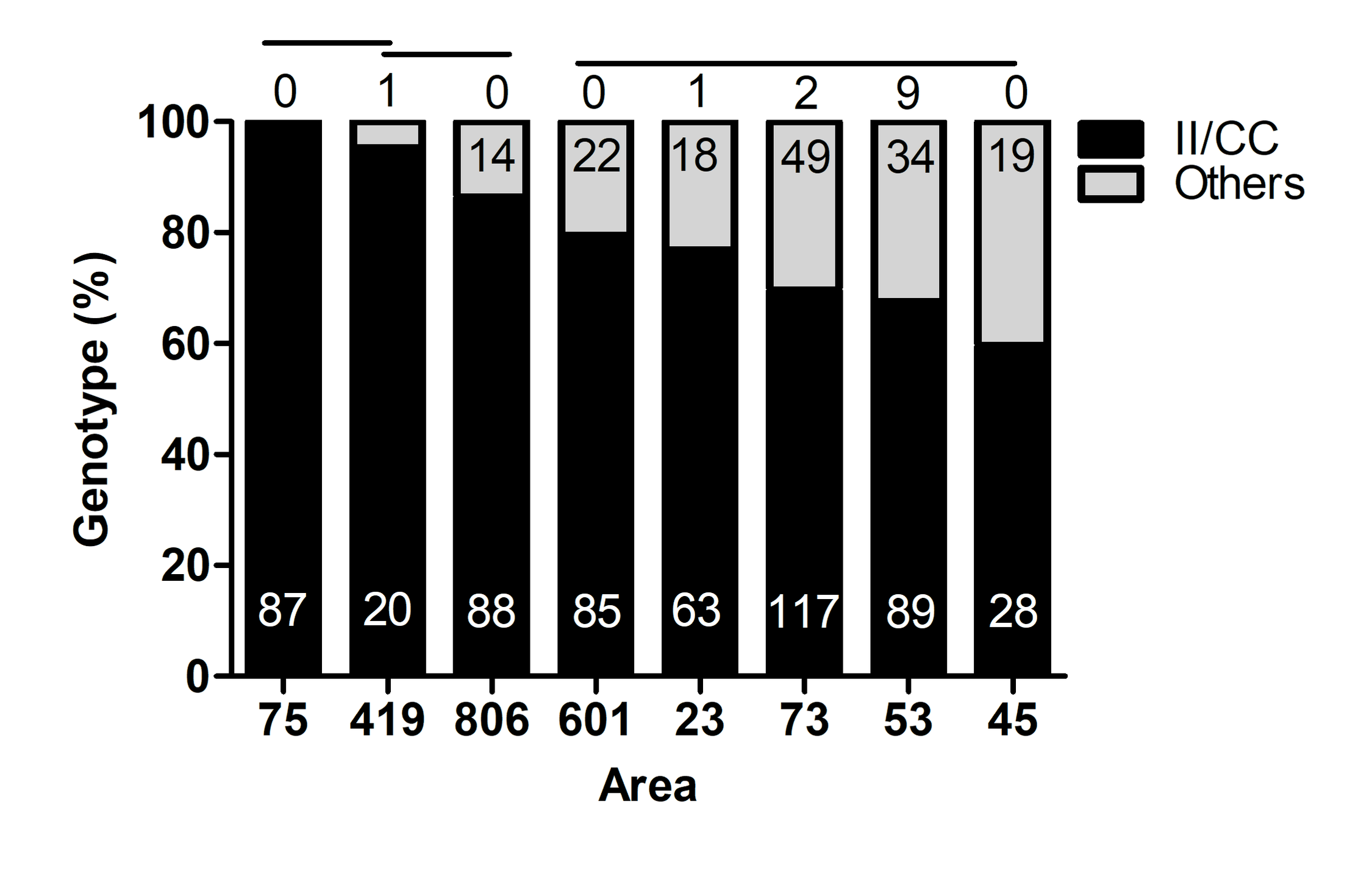

Supplement: S3 Fig — The numbers in the graph refer to the number of mosquitoes analyzed for each area: in black above each bar are the number of susceptible (VV/FF) mosquitoes; white numbers on black bars are double homozygous resistant (II/CC) females; in black on grey areas are the number of mosquitoes of all other genotypes that were not VV/FF or II/CC. Horizontal lines above bars indicate the areas which are not significantly different (P < 0.05). (TIF) [file pntd.0009833.s003.tif]

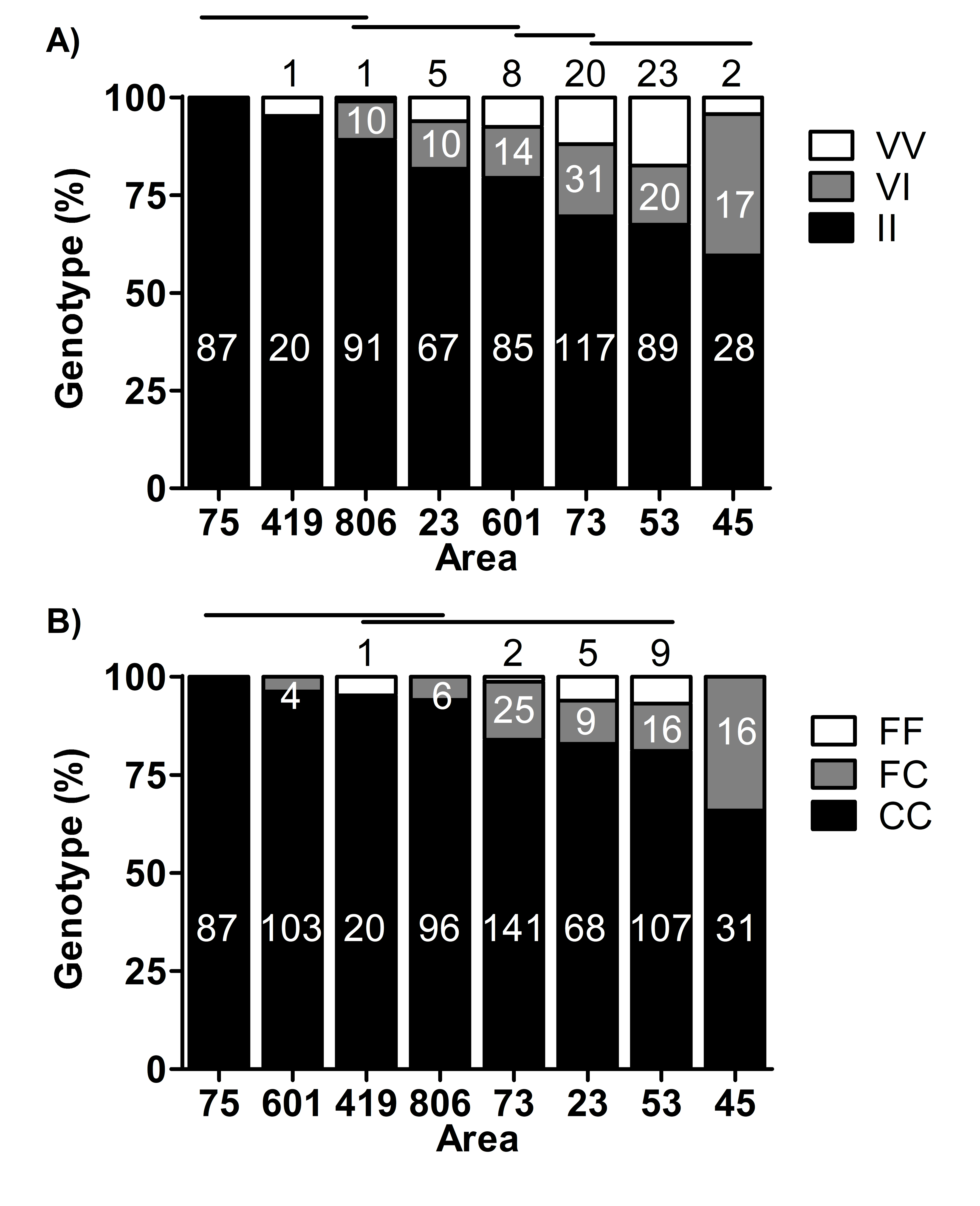

Supplement: S4 Fig — Percentage of the (A) V1016I mutation and (B) F1534C mutation in females of Ae aegypti. Significant differences were detected among the proportion of genotypes observed across all areas for each mutation site analyzed independently (Chi-square; P < 0.0001 for each, A and B panels). Horizontal lines above bars indicate areas in which the percentage of genotypes are not significantly different (P < 0.05). Numbers above bars indicate the number of susceptible field-collected mosquitoes (either VV or FF, respectively) detected. Numbers in white on black bars are double homozygous resistant (II or CC) females; in white on grey areas are the number of heterozygous females (VI or FC, respectively). (TIF) [file pntd.0009833.s004.tif]

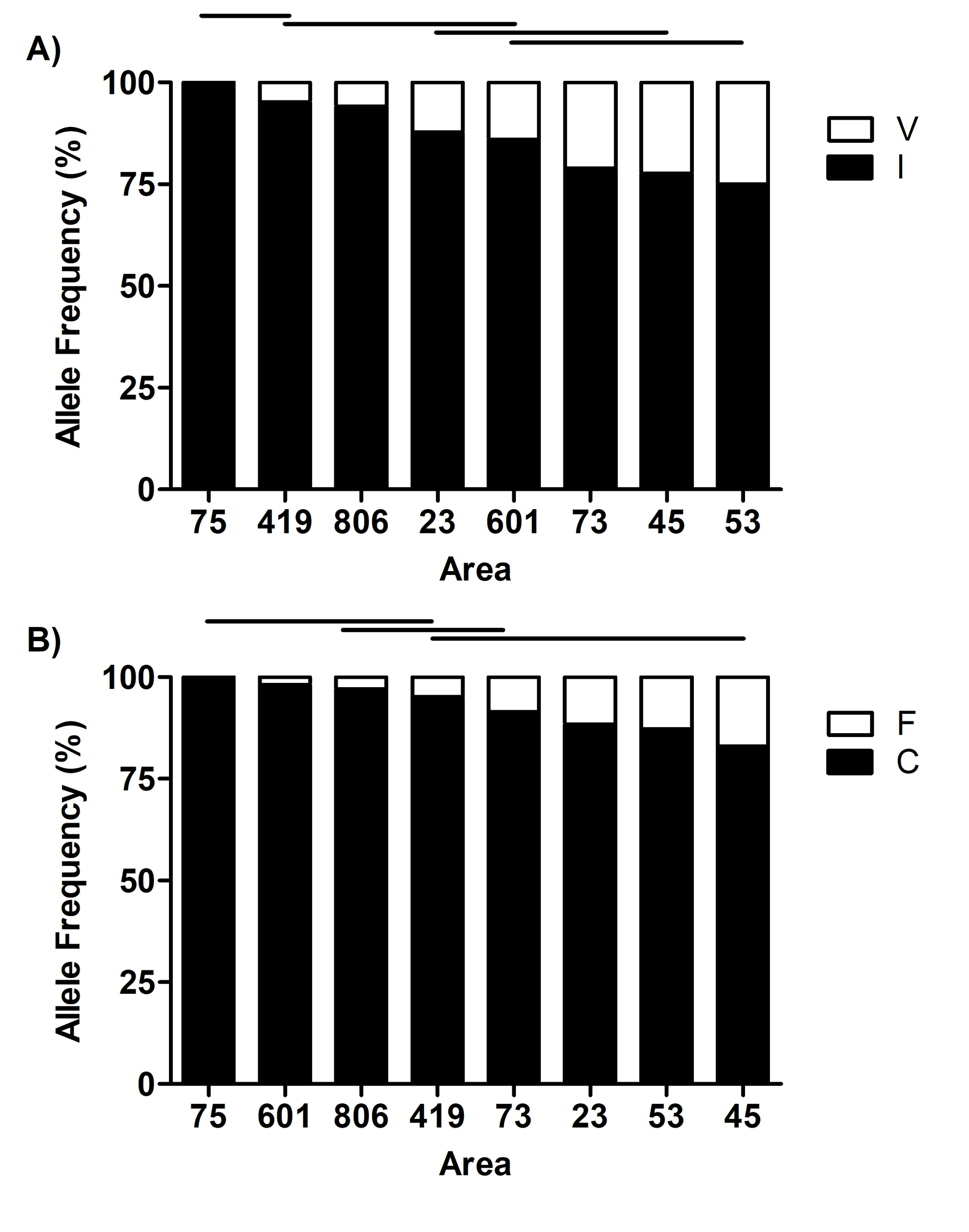

Supplement: S5 Fig — Percentage of the (A) V and I alleles at position 1016, and (B) F and C alleles at position 1534. Significant differences were detected across all areas between the proportion of the two alleles V and I (A), or F and C (B) for each mutation site (Chi-square; P < 0.0001 for each panel). Horizontal lines above bars indicate the areas which are not significantly different (P < 0.05). In each panel, note that areas are organized in order of decreasing frequency of the resistant allele. Note that areas are grouped differently for their similarity in the frequency of each of the resistant alleles. (TIF) [file pntd.0009833.s005.tif]

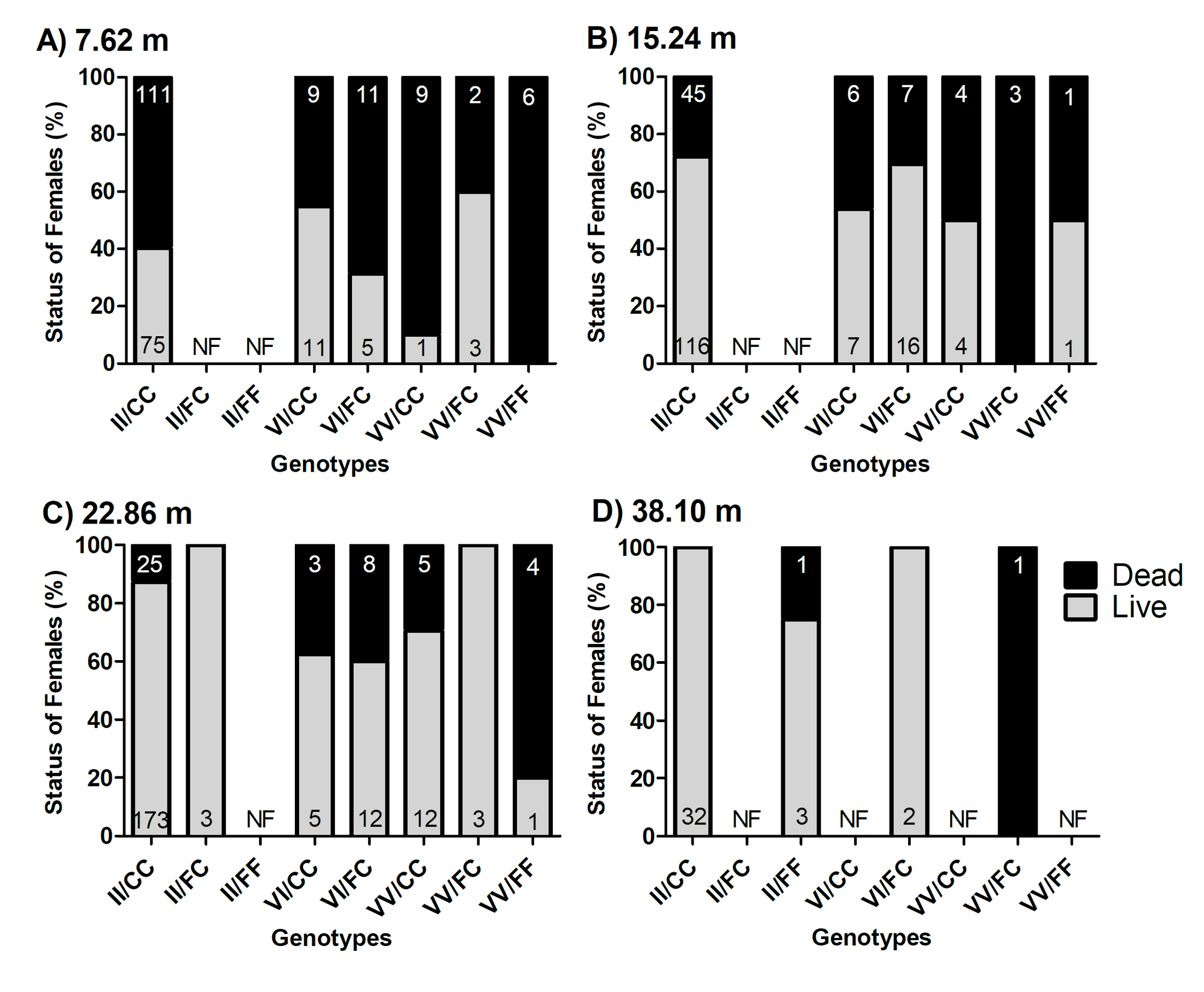

Supplement: S6 Fig — Panels show results of each of the tested distances from the Permanone 31–66 application source, as follows: (A) 7.62 m, (B) 15.24 m, (C) 22.86, and (D) 38.1 m. In black on the gray zone in each bar is the number of genotyped mosquitoes that survived, and in white in the black zone are the number of genotyped mosquitoes that perished. NF: none found. The data as shown here could not be statistically analyzed due to the lack of individuals for some genotypes in different areas and for some distances. This figure supplements data in Fig 6 in the main text. (TIF) [file pntd.0009833.s006.tif]

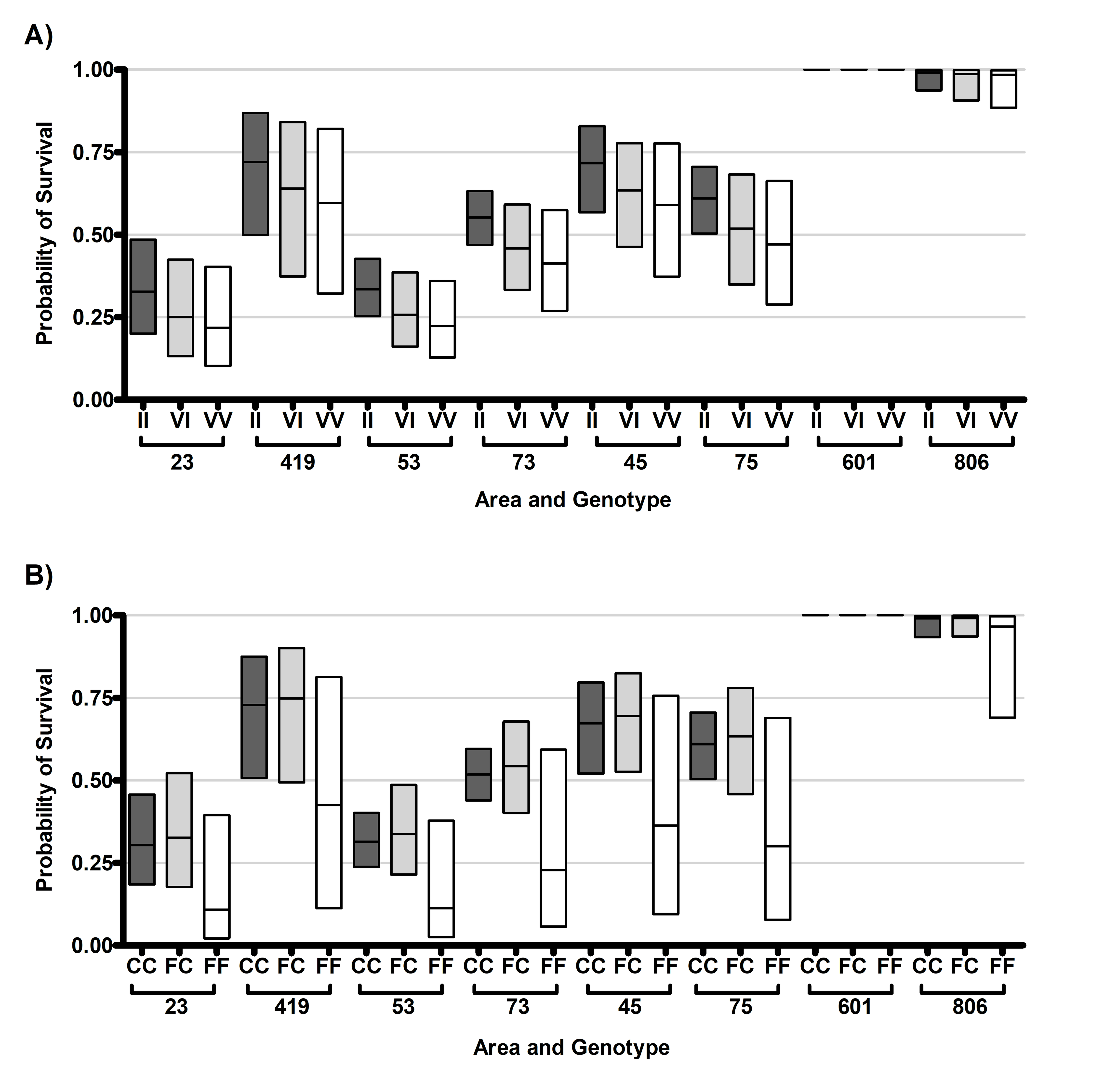

Supplement: S7 Fig — Probability of survival of females of Ae. aegypti carrying the (A) V1016I mutation or (B) the F1534C mutation. The boxes’ middle lines represent the predicted probability of survival obtained by logistic regression analysis. The top and bottom values of the boxes are the upper and lower 95% CIs. The overall model fit (logistic regression, P < 0.0001). An asymptomatic Wald’s procedure was used to obtain the CIs for the probability of survival. (TIF) [file pntd.0009833.s007.tif]
